# Enzymatic and Microbial Electrochemistry: Approaches and Methods

**DOI:** 10.1021/acsmeasuresciau.2c00042

**Published:** 2022-08-29

**Authors:** Giada Bedendi, Lilian D. De Moura Torquato, Sophie Webb, Cécile Cadoux, Amogh Kulkarni, Selmihan Sahin, Plinio Maroni, Ross D. Milton, Matteo Grattieri

**Affiliations:** † Department of Inorganic and Analytical Chemistry, Faculty of Sciences, University of Geneva, Quai Ernest-Ansermet 30, 1211 Geneva 4, Switzerland; ‡ National Centre of Competence in Research (NCCR) Catalysis, University of Geneva, Quai Ernest-Ansermet 30, 1211 Geneva 4, Switzerland; § Dipartimento di Chimica, Università degli Studi di Bari “Aldo Moro”, via E. Orabona 4, Bari 70125, Italy; ∥ IPCF-CNR Istituto per i Processi Chimico Fisici, Consiglio Nazionale delle Ricerche, via E. Orabona 4, Bari 70125, Italy

**Keywords:** biocatalysts, bioelectrochemical systems, cyclic voltammetry, hyphenated electrochemical techniques, electrochemical quartz crystal microbalance, fluorescence, mediated electron transfer, spectroelectrochemistry, synthetic biology

## Abstract

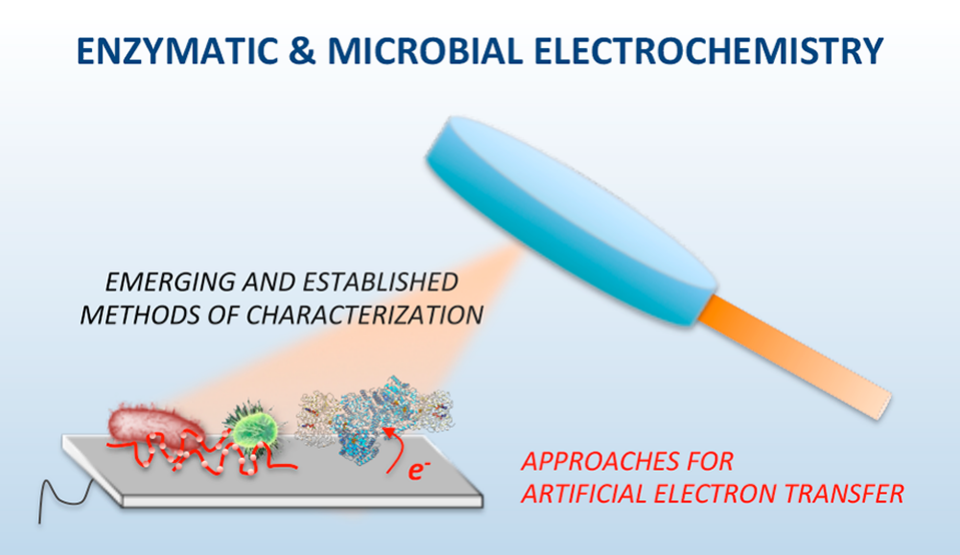

The coupling of enzymes and/or intact bacteria with electrodes has been vastly investigated due to the wide range of existing applications. These span from biomedical and biosensing to energy production purposes and bioelectrosynthesis, whether for theoretical research or pure applied industrial processes. Both enzymes and bacteria offer a potential biotechnological alternative to noble/rare metal-dependent catalytic processes. However, when developing these biohybrid electrochemical systems, it is of the utmost importance to investigate how the approaches utilized to couple biocatalysts and electrodes influence the resulting bioelectrocatalytic response. Accordingly, this tutorial review starts by recalling some basic principles and applications of bioelectrochemistry, presenting the electrode and/or biocatalyst modifications that facilitate the interaction between the biotic and abiotic components of bioelectrochemical systems. Focus is then directed toward the methods used to evaluate the effectiveness of enzyme/bacteria–electrode interaction and the insights that they provide. The basic concepts of electrochemical methods widely employed in enzymatic and microbial electrochemistry, such as amperometry and voltammetry, are initially presented to later focus on various complementary methods such as spectroelectrochemistry, fluorescence spectroscopy and microscopy, and surface analytical/characterization techniques such as quartz crystal microbalance and atomic force microscopy. The tutorial review is thus aimed at students and graduate students approaching the field of enzymatic and microbial electrochemistry, while also providing a critical and up-to-date reference for senior researchers working in the field.

## 1 Introduction

### 1.1 Enzymatic and Microbial Electrochemistry

Enzymes represent the majority of the natural catalytic machinery enabling several complex reactions in living organisms. Specifically, oxidoreductases are enzymes that catalyze the transfer of electrons between two (or more) substrates, making them arguably the most interesting and commonly utilized enzymes in electrochemical systems on electrode surfaces. The features offered by oxidoreductase enzymes are (i) high selectivity toward their substrates and (ii) good activity under ambient conditions (ambient temperature, near-neutral pH). There is also substantial interest in redox-active proteins (those not catalyzing reactions) at electrode surfaces, although these will not be discussed extensively in this tutorial review as similar principles for their application in bioelectrochemical systems apply. Conversely, intact and viable bacteria provide other unique features: (i) a broad substrate scope, (ii) replication and adaptation capabilities, and (iii) the catalytic machinery for complex multistep reactions. Accordingly, they can be seen as complex biofactories where the presence of several enzymes provides a sophisticated enzymatic machinery that enables critical reactions such as the oxidation of organic substrates coupled to hydrogen production, or water splitting coupled to CO_2_ reduction.

When coupling enzymes and bacteria with electrodes, an underlying objective is to successfully transfer electrons between the abiotic component (the electrode) and the redox-active center(s) (cofactors) of the enzymes (either isolated or inside the bacterial cells), thus accomplishing a “connection”, also defined as “electrical wiring”, between electrodes and redox-active cofactors.

Accordingly, the tutorial review begins by briefly introducing the various biocatalysts utilized in bioelectrochemical systems and their applications, with a following section dedicated to the electron transfer process at the biotic/abiotic interface. The approaches utilized to accomplish such process are presented, including direct and mediated (extracellular) electron transfer, before discussing the characterization of enzymatic and microbial electrochemical systems. The state-of-the-art techniques utilized in enzymatic and microbial electrochemistry are considered, comparing their advantages, the insights that they provide, and the challenges in their application to study such systems. The tutorial review closes with an outlook on the various approaches and methods for the future development and characterization of bioelectrochemical systems.

### 1.2 Biocatalysts for Enzymatic and Microbial Electrochemical Systems

Redox proteins and enzymes consist of a polypeptidic protein structure within which one or multiple redox-active cofactors can be housed. These cofactors can be coordinated metallic cofactors such as FeS clusters (ranging from [2Fe-2S] to the [7Fe-9S-C-Mo/V/Fe] cofactor of nitrogenases) or hemes or diffusive/tightly bound organic molecules such as nicotinamide adenine dinucleotide (NAD, *E*^0^′ = −0.32 V vs SHE) or pyrroloquinoline quinone (PQQ, *E*^0^′ = +0.07 V vs SHE).^1,2^ The mechanisms of these enzymes include at least one electron transfer event associated with the conversion of the substrate(s) into product(s). One example of a “simple” redox enzyme (in terms of structure and the number of redox-active cofactors) is peroxidase (formally peroxide reductase), which can employ a single heme cofactor for the 2*e*^–^/2H^+^ reduction of peroxides, yielding water (H_2_O) in the case of hydrogen peroxide (H_2_O_2_) reduction. On the other hand, the electron-bifurcating heterodisulfide reductase found in methanogenic archaea contains a total of 28 FeS clusters and two flavin adenine dinucleotide (FAD) cofactors, which together facilitate the thermodynamically uphill (with respect to equilibrium potentials) transfer of electrons for the reduction of carbon dioxide in methanogenesis.^3,4^ Such large differences in the structures and functions of redox enzymes typically precludes the design of a universal electrode architecture for electron transfer with redox enzymes.

While focusing on bacteria, they contain several enzymes, enabling their variegate and complex metabolisms. Among others, it is possible to distinguish photosynthetic and non-photosynthetic bacteria depending on their capability to utilize sunlight as an energy source by means of a photosynthetic apparatus. Photosynthetic purple bacteria are one of the first species that appeared on Earth (about 3.7 billions years ago) that later led to the evolution of cyanobacteria and the oxygenation of Earth’s atmosphere.^5,6^ Other bacteria evolved enzymatic machinery, allowing them to utilize complex substrates as electron donors or acceptors, and recent reports have shown that bacteria can even biodegrade chemically stable perfluorinated carboxylic acids.^7^ One critical point is that bacteria have external membranes (the numbers of membranes varies depending on the type of bacteria, i.e., Gram-negative or Gram-positive) that constitute a physical separation of the enzymes in the cytoplasm or the membrane-bound proteins from the external environment. Accordingly, accomplishing the electrical wiring of intact bacteria with electrode surfaces is one of the major challenges in microbial electrochemistry.^8−10^

### 1.3 Applications of Enzymatic and Microbial Electrochemical Systems

**Figure 1:**
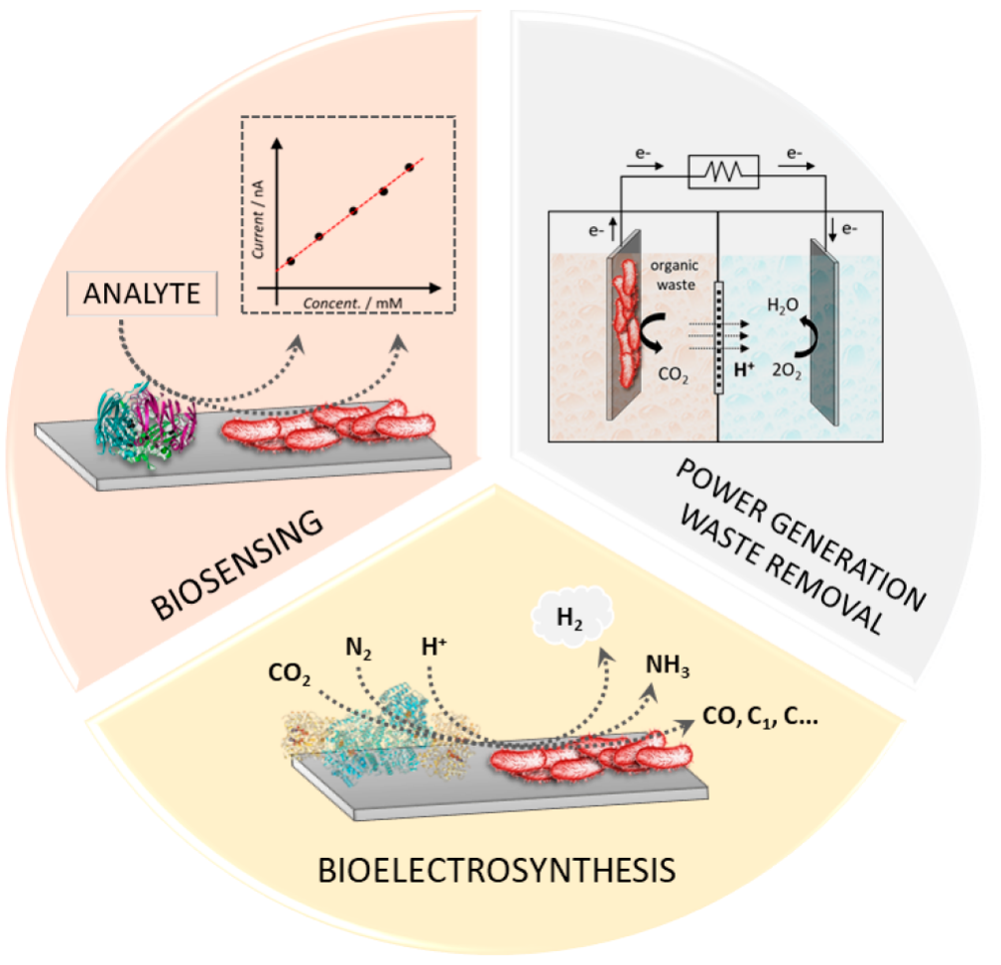
Scheme of different applications of enzymatic and microbial electrochemical systems, including the development of biosensors for the monitoring of medically relevant analytes or toxic compounds, the local generation of micropower with simultaneous waste removal, and the development of bioelectrosynthetic factories.

Figure 1 shows the three most common applications of enzymatic and microbial electrochemical systems. Perhaps, the most widely investigated use of enzymes on electrodes has been for biomedical applications, with enzyme-based amperometric biosensors providing a current that is related to the concentration of an analyte (the enzymatic substrate). The most famous example has been the development of glucose biosensors, in which a glucose-oxidizing enzyme (immobilized at the surface of an electrode) oxidizes glucose by 2*e*^–^ to gluconolactone.^11^ This is the predominant technology employed in commercially available glucometers used by diabetes patients. Expanding this further, it is possible to use these same substrate-oxidizing electrodes within enzymatic fuel cells (EFCs). Such devices depend on redox enzyme catalysis on at least one electrode surface, with traditional metal electrodes typically performing the circuit-closing reaction (i.e., the reduction of O_2_ to H_2_O on Pt cathodes). Importantly, redox enzymes can also be utilized on cathodes for reactions such as the 4*e*^–^ reduction of O_2_ to H_2_O. Full EFCs thereby employ enzymes on both the anodes and cathodes, with examples being glucose/O_2_ and H_2_/O_2_ fuel cells.^12−15^

Given that enzymes are known to be able to activate stable small molecules under mild conditions, there is also significant interest in the electroenzymatic synthesis of value-added products.^16−18^ For example, hydrogenases are metalloenzymes that catalyze both H_2_ oxidation and H^+^ reduction, reversibly; hydrogenase-containing electrodes are therefore able to produce H_2_ from electrical energy in aqueous solutions.^19−21^ Similarly, nitrogenases have gained much interest as the only enzyme able to reduce kinetically inert dinitrogen (N_2_) into ammonia (NH_3_), a value-added product which is the building block for many nitrogen-based compounds (fertilizers, amino acids, DNA bases, polymers, etc.).^22^ Carbon dioxide (CO_2_)-reducing enzymes have also gained interest over the last decades because of their ability to reduce CO_2_ into C-based value-added fuels such as carbon monoxide (CO) or formate.^23,24^ Finally, it is also possible to combine these enzymes within EFCs, where electrical energy and value-added products can be produced simultaneously. However, the reductive enzymes outlined here are typically associated with reactions with relatively low *E*^0^ (*E*^0^′_2H^+^/H_2__ = −0.41 V vs SHE, *E*^0^′_CO_2_/HCOOH_ = −0.42 V vs SHE, *E*^0^′_N_2_/2NH_3__ = −0.14 V vs SHE), which then requires a low-potential anodic system for the EFC to be truly galvanic. Nevertheless, a hydrogenase anode was combined with a nitrogenase cathode to yield a H_2_-oxidizing and N_2_-reducing EFC with an open circuit potential (OCP) of 0.23 ± 0.03 V. 60 mC of charge was drawn from this device, while simultaneously producing NH_3_ with 26.4% Faradaic efficiency.^25^

Concerning the use of bacteria on electrodes, the most widely investigated application has been the wiring of electroactive bacteria (species that are naturally capable to transfer electrons across the cellular membrane due to endogenous electron transfer pathways) for power generation and environmental remediation purposes.^26,27^ Specifically, *Shewanella oneidensis* (*S. oneidensis*) and *Geobacter sulfurreducens* (*G. sulfurreducens*) are the two species with the most detailed extracellular electron transfer pathways described to date. These two species have been consistently utilized at the anode of microbial fuel cells, achieving relatively high current and power densities (about 4 mA cm^–2^ and 0.6 mW cm^–2^, respectively).^28^ More recently, focus has been posed on the use of microbial electrochemical systems for biosensing^29^ and bioelectrosynthesis.^30^ In the case of biosensing applications, the current or power output of the microbial electrochemical system is correlated to the concentration of an analyte of interest, and self-powered biosensing devices have been obtained when utilizing complete microbial fuel cells.^31,32^ Regarding the possibility to obtain microbial bioelectrofactories, recently, cyanobacterial cells were engineered to express the electron transfer pathway of *G. sulfurreducens*, which enabled them to utilize a cathode as the electron donor to accomplish N_2_ reduction to NH_3_.^33^

Finally, bacteria and enzymes-based electrodes have been utilized together in the same electrochemical system to obtain enzyme/microbial hybrid systems, where microbial anodes oxidizing various substrates are couple to enzymatic cathodes accomplishing the oxygen reduction reaction for high-performance devices.^34,35^ These hybrid systems have been further expanded to have microbial fuel cells charging an abiotic electrode that is subsequentially coupled to an enzymatic electrode for CO_2_ reduction.^36^

## 2 Principles of Bioelectrochemistry

Semiclassical Marcus theory for electron transfer in nonadiabatic systems has been extensively applied to the study of electron-transferring proteins and enzymes:^37^
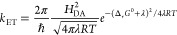
where the electron transfer rate constant (*k*_ET_) is dependent on the variables (i) temperature, (ii) thermodynamic driving force of the reaction (Δ_r_*G*^0^), (iii)nuclear reorganization energies (λ), and (iv) matrix coupling element (*H*_DA_, also commonly referred to as their electronic coupling). Importantly, *H*_DA_ exponentially decays with an increase in distance (*r*_DA_) between electron transfer partners, and depends on the medium in between (i.e., amino acids, solvent, etc., given as an electron transfer decay parameter “β”):
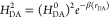
where *H*_DA_^0^ reflects the electronic coupling of the donor and acceptor when they are in contact (*r* = 0). Even though electrode-adsorbed proteins are known to deviate from Marcus theory of electron transfer (with conformational reorganizations thought to become rate-limiting to heterogeneous electron transfer), it arguably remains intuitive that the distance between an electrode and the redox-active cofactor of an enzyme should be minimized to maximize heterogeneous *k*_ET_.^38^ This is even more intuitive for the case of intact bacteria-electrode systems, where the electron-transferring enzymes responsible for the exchange of electrons are located inside the bacterial cells, thus separated from the electrode surface by several nm (normally more than 30 nm).^39^

**Scheme 1:**
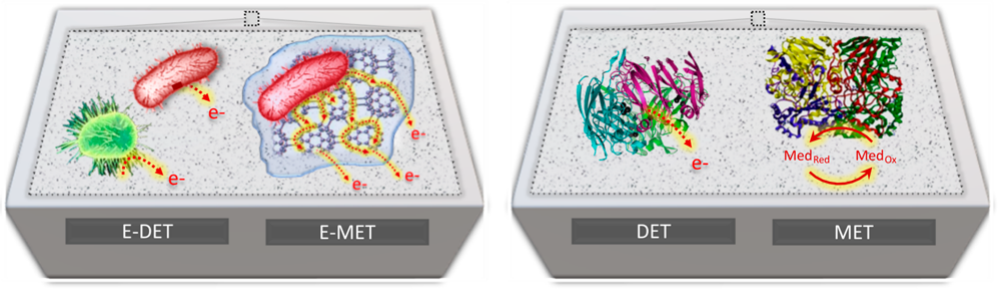
Electron Transfer Pathways for Microbial (Left) and Enzymatic Electrodes (Right) E-DET refers to the EET of electrons through microbial nanowires or membrane-bound proteins. E-MET refers to the EET by means of diffusible or polymer bound-redox mediators. DET refers to the direct transfer from the redox-active sites of an enzyme to the electrode surface, while MET refers to the electron transfer through artificial redox mediators.

Below, the major electron transfer pathways are briefly presented, both for enzymatic and microbial electrochemical systems. In this tutorial review, we have utilized the classification of electron transfer pathways that is more commonly adapted in bioelectrochemistry, with direct electron transfer (DET) being obtained when an enzyme transfers electrons directly with an electrode. On the contrary, mediated electron transfer (MET) refers to systems requiring an exogenous, artificially added, electron mediator that shuttles electrons between enzymes and electrodes. For the case of intact bacteria, the electron transfer process is more properly defined as “extracellular electron transfer” (EET),^40^ as electrons need to cross the bacterial membrane, and EET is further divided into direct (E-DET) and mediated EET (E-MET), as shown in Scheme 1.

### 2.1 Direct Electron Transfer (DET)

There are both advantages and disadvantages to DET configurations.^12,41,42^ From an applied perspective, DET can simplify the experimental setup and therefore lower the cost of an enzymatic device if an enzyme can spontaneously adsorb to an electrode surface (electrostatic, π–π stacking, π–cation interactions) and undergo efficient electron transfer.^43,44^ In this case, an additional electron mediator may also not be necessary, although this approach becomes more costly if tailored electrode/enzyme modifications are necessary to achieve DET. An important benefit that is afforded by DET is the ability to interrogate the redox-active cofactors of an enzyme, paving the way to the possibility of determining their reduction potentials (*E*^0^′). Furthermore, DET provides a handle by which relationships between thermodynamic (driving force) and kinetic properties (catalytic rate constants or affinities) of enzymes can be studied.^45^

One potential disadvantage to DET for practical applications is that only a single monolayer of enzyme is expected to be electronically wired to the electrode, and the quantity of electroactive enzyme (and thus the current) is therefore somewhat limited. One strategy to overcome low current densities is to use conductive, stable and hydrophobic nanoparticles (NPs) and carbon nanotubes (CNTs). For instance, CNTs have been used in the presence of pyrene based anchoring compounds based on π–π stacking interactions.^46^ Complementary strategies using surfactants, hydrogels or polymers (PEG, chitosan, alginate, etc.) provide a matrix that can also increase stability over time.^44,47^ In addition, the use of conductive porous materials such as indium–tin oxide (ITO) or metal–organic frameworks (MOFs) permit larger surface areas, and therefore larger enzyme loadings/coverages on electrode surfaces.^48,49^ Despite adsorption being the simplest way to modify electrode surfaces, this is often not successful for many enzymes because of factors such as (i) rapid denaturation, (ii) low film/enzyme stability over time, and (iii) enzyme leaching/film loss. As discussed above, adsorption should also not inhibit electron transfer between the enzyme and electrode. Covalent anchoring of enzymes enables some of these issues to be overcome, with amide bond formation through coupling carboxylic acid-functionalized surfaces to protein surface lysine residues, or by cross-linking glutamate and aspartate residues from protein surfaces to amine-functionalized surfaces. This coupling can be performed with peptide coupling agents, with EDC/NHS being extensively used due to its stability in water and mild operational conditions.^50,51^ Another possibility is to modify the electrode surfaces by electrografting diazonium salts, which provides a universal technique to introduce a large diversity of chemical functions on the surface of the electrode.^52^

One prominent and versatile strategy that has been extensively employed to optimize DET is the use of thiol-containing species, which form self-assembled monolayers on gold electrodes.^53,54^ The main advantage of this technique is adjustable lengths of chains and the possibility to introduce different terminal end groups. The direct anchoring of enzymes on gold is also possible via the reaction of solvent/surface-exposed cysteine (sulfhydryl-) residues on proteins. Because of the comparatively lower occurrence of cysteine in proteins vs lysine residues, this approach is more specific than amide bond formation (discussed above). Further, cysteine residues can also be inserted in or relocated to strategic positions (i.e., close to a redox-active cofactor) via site-directed mutagenesis. This method will be discussed in depth below. It has also been proposed that nonspecific enzyme adsorption on electrodes leads to a distribution of heterogeneous *k*_ET_, which can complicate the interpretation of thermodynamic and kinetic parameters.^55^ Site-specific covalent adsorption (i.e., by exploiting cysteine residues) is considered one potential way to overcome this limitation. It is also possible to exploit surface cysteine chemistry by immobilizing Michael acceptors such as maleimides on electrode surfaces, which then undergo thiol-maleimide Michael additions.^56^ This ligation method is widely used in the field of chemical biology because of the fast kinetics and high selectivity of this reaction at neutral pH. The value added by this method compared to protein adsorption in self-assembled monolayers (SAMs) or on gold surfaces is the formation of a covalent bond between the thiol group of the cysteine and the maleimide, which is not sensitive to low potential reductive cleavage (and release), such as in the case of Au–thiol bonds.

### 2.2 Mediated Electron Transfer (MET)

MET is an artificial electron transfer approach that employs redox-active species (either diffusive or bound to polymer backbones) to shuttle electrons between enzymes and electrodes.^57^ This approach allows overcoming the limitations due to the redox-active cofactor being distant from the surface, and makes specific protein adsorption no longer a requirement. Furthermore, MET extends the bioelectrode design to enzymes in solution, since only the mediator is required to interact with the electrode surface. For applied enzymatic electrochemistry, this configuration provides the ability to exploit flow chemistry techniques, or to have large quantities of biocatalyst present in the cell (compared to a DET monolayer), although the separation of products from the enzyme and mediator is presumably required postprocessing. Important requirements for an appropriate electron mediators include (i) having good electrochemical reversibility (stable in both reduced and oxidized state), (ii) having good solubility, (iii) having fast rates of electron transfer (with the enzyme and the electrode), (iv) and not adversely affecting enzymatic activity.

As a rule of thumb, the electroenzymatic oxidation (e.g., glucose oxidation) of a substrate typically requires an electron mediator with an *E*^0^′ that is more positive than the *E*^0^′ of the enzyme’s redox-active cofactor and the substrate/product (with the opposite being true for reductive electroenzymatic reactions, e.g., H^+^ reduction), as dictated by
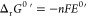
where *n* is the number of electrons in the electron transfer process, *F* is Faraday’s constant (96,485 C mol^–1^), and *E*^0^' is the reduction potential of the reaction. This can be extended to MET by considering the reduction potentials of the substrate/product (the enzymatic reaction, *E*^0^'_S/P_) and the oxidized/reduced electron mediator (*E*^0^'_O/R_) to yield^58^
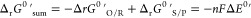
and
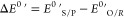
It is important to keep the relative positions in equilibrium (Δ*G*) in mind when considering electron mediators: for example, the electron mediator methylviologen (*E*^0^′ = −0.45 V vs SHE) can effectively mediate enzymatic H^+^ reduction and H_2_ oxidation (*E*^0^′ = −0.41 V vs SHE). This principle previously enabled the use of methylviologen in both the anodic and cathodic compartments of the H_2_/N_2_ EFC outlined above.^25^

A breakthrough advance for enzymatic MET was the development of redox polymers.^59,60^ In such redox mediating systems, suitable electron mediators are first chemically grafted to water-soluble polymeric backbones and the resulting material can following be mixed with a solution of enzyme and drop-casted onto an electrode surface (possibly in the presence of a chemical cross-linker). The result is an enzyme/polymer film that is (i) confined to the electrode surface and (ii) contains a nondiffusing electron mediator, which subsequently permits MET across the entire redox polymer/enzyme film with electrons hopping across the mediator moieties. Redox polymer/enzyme composites have found extensive use in blood glucose monitoring. A recent major advance in redox polymers was the development of a system that simultaneously enabled the electroenzymatic oxidation of H_2_ by hydrogenase, while simultaneously protecting this enzyme from O_2_ deactivation (using the same H_2_ enzymatic oxidation reaction).^61^

### 2.3 Extracellular Direct and Mediated Electron Transfer

As previously introduced, interfacing intact bacteria with electrodes is complicated by the presence of the cytoplasmic membrane (often referred to as inner membrane) and other additional membrane layers (i.e., peptidoglycan, outer membrane), which are physical barriers surrounding the bacterial cells acting as insulators. However, some bacterial species were forced to evolve dedicated electron transfer pathways as they found themselves in environments with few or no soluble electron acceptors available, thus having to utilize insoluble electron acceptors located outside of their membranes (i.e., iron oxides).^40,62^ This E-DET differs from the DET discussed for enzymatic systems due to the various intermediate electron carriers at plays between the redox enzymes and the electrodes. The two better-understood E-DET pathways are the ones of *S. oneidensis* and *G. sulfurreducens*. Excellent studies describe in detail the electron hopping mechanism across various cytochromes located in the external membranes of *Shewanella* species,^63^ where the electrons are shuttled across more than 25 nm of insulating space.^64^*Geobacter* species exhibit a similar electron transfer pathway using porin-cytochrome trans-outer membrane protein complexes,^65^ while also utilizing conductive nanowires that transfer charge by π–π stacking, in a similar fashion to carbon nanotubes.^66^ Additionally, *Shewanella* performs E-MET by self-secreting riboflavin, an endogenous redox mediator that acts as a diffusible shuttle from the redox-active sites to the electrode surface.^67^*Shewanella* is not the only species known for producing endogenous mediators, with recent reports available also for other bacteria, including cyanobacteria.^68−70^ Furthermore, the modification of the electrode surfaces to facilitate E-MET via endogenous mediators have also been investigated, i.e., with the use of carbon nanotubes.^71^ E-MET is also possible through exogenous diffusible redox mediators, which enabled the use of various photosynthetic bacteria for biophotoelectrodes development.^72,73^

The use of redox polymers has proven particularly effective for E-MET, with pioneering works employing osmium-based redox polymers to wire several bacterial species.^74−76^ More recently, bioinspired redox polymers that utilize redox moieties similar to the natural electron mediators found in bacteria, such as quinone molecules, have been employed successfully wiring Gram-positive and Gram-negative bacteria.^77^ Remarkably, such polymers enabled a consistent enhancement of electron transfer also for the electroactive bacterium *S. oneidensis*.^78^ With the aim to provide both artificial redox mediation and adhesive properties to maintain the bacteria on the electrode surface, redox-adhesive polymeric matrices based on polydopamine have been recently developed for E-MET with various bacteria.^79−81^ As photosynthetic bacteria have intrinsic limitations to the EET due to the location of the photosynthetic apparatus inside the inner membrane, the reader is referred to a recent review discussing this aspect and presenting the various artificial redox-mediating systems specifically developed for this class of bacteria.^82^

### 2.4 Synthetic Biology for Electrochemistry

#### 2.4.1 Protein Engineering: Affinity Tags, Cysteine Residue Chemistry, and Unnatural Amino Acids

Given that *most* enzymes/proteins have not been evolved to transfer electrons with nonproteinogenic surfaces, below are discussed some prominent and emerging protein engineering approaches that have been developed to optimize enzymatic electrochemistry.

One approach utilizes the recombinant insertion of an affinity polypeptide tag within the primary structure of a target protein to either its N- or C-terminus that provides a specific and simplified strategy for protein purification. Perhaps the most-used affinity tag is poly(histidine)_(5–8)_ (His-tag), which then typically displaces two aqua ligands of a coordination complex such as [Ni(NTA)(OH_2_)]^2+^ (NTA = nitrilotriacetic acid). The NTA ligand is covalently bound to a solid phase, and alternative first-row *d*-block metal cations can be used in the place of Ni^2+^ (Fe^2+^/Co^2+^/Zn^2+^).^83^ The repeating histidine sequence exploits the chelate effect to provide greater affinity than other individual solvent-exposed histidine residues. The same principle can be exploited to anchor enzymes to electrode surfaces by their His-tags, where gold electrodes have been modified with Ni-NTA SAMs for the immobilization of His-tagged PQQ-dependent aldehyde dehydrogenase (PQQ-AlDH)^84^ and horse-heart cytochrome *c* (cyt *c*).^85^ By inserting His-tag to either the N- or C-term of each of PQQ-AlDH’s three subunits, six different variations were obtained with one favorably orientated on the electrode showing 6.6-fold higher DET efficiency.^84^ In the case of cyt *c*, a C-terminal His-tag containing variant showed the most efficient DET because of minimized electron transfer distances.^85^ Also, Ganesana et al. functionalized the surface of graphite screen-printed electrodes with nickel oxide nanoparticles for the immobilization of His-tagged acetylcholinesterase obtaining a biosensor with improved sensitivity.^86^ Recently, Minteer’s research group also introduced another approach for the immobilization of His-tagged proteins on carbon electrodes where a Ni-chelated-Lys_3_Asp polypeptide linked to a pyrene moiety (for π–π stacking with carbon surfaces) was produced by solid-phase peptide synthesis.^87^ The His-tagged MoFe protein of nitrogenase was subsequently immobilized for MET with adding methylviologen, without the need to entrap the MoFe protein within a polymeric support layer. However, the use of His-tags has several disadvantages, including the limited modification to either the C- or N-termini of protein subunits.^51,88,89^

**Figure 2:**
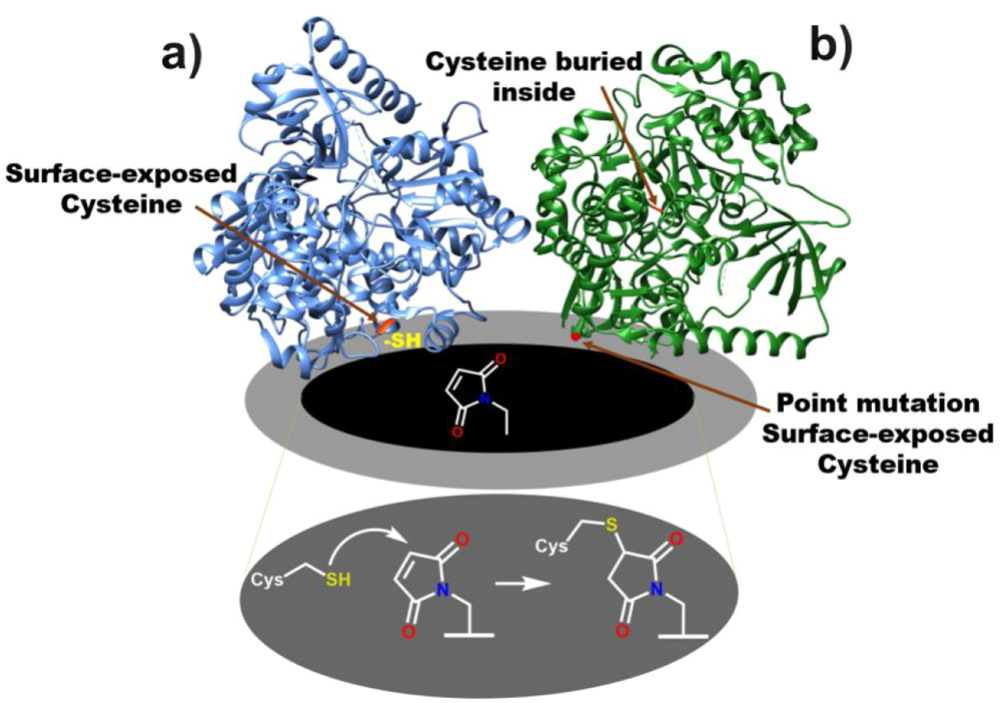
(a) Metalloprotein with a surface-exposed cysteine can readily react with a scaffold incorporated on the electrode, such as maleimide. (b) In case of a protein having a cysteine residue not exposed on the surface, a point mutation can help to introduce a cysteine residue at the suitable position.

Another approach involves targeting specific amino acids (i.e., lysines, cysteines, aspartates) on the surface of a protein for chemical conjugation. For example, the primary amines of lysines can be targeted for amide bond formation with the use of reagents such as EDC/NHS (as mentioned above). However, a protein’s surface typically has multiple solvent-exposed lysine residues, neutralizing the possibility for oriented protein immobilization. Among all the residues on the proteome, cysteine is one of the most nucleophilic and conserved, making it by far the most reactive residue on any protein, yet the least abundant residue.^90^ This high reactivity of cysteine has been extensively utilized for various purposes, including drug-development strategies,^91−93^ bioconjugation strategies, development of various probes for imaging of biological species,^94,95^ and proteomics.^96^ Many of these bioconjugation reactions can be extended to enzymatic electrochemistry. For example, these Michael acceptors undergo 1–4 cycloaddition^97^ (Figure 2a), thus the electrodes can be functionalized with groups that readily react with cysteine thiols, such like maleimide Michael acceptors.^98,99^

In certain proteins, the reactive cysteine residues may be buried inside, i.e., they may not be accessible to the solvent or other reactive molecules, and thus not be readily available on the surface for conjugation (Figure 2b).^100^ In such cases, using molecular biology techniques, like site-directed mutagenesis, a cysteine residue can be introduced on the protein surface in a suitable position.^99^ The protein structure or its activity are not particularly affected by point mutations of residues not directly involved in enzymatic processes, making them the ideal way to introduce the cysteine residue in a more suitable position for efficient immobilization on the electrodes. Meneghello et al. exploited site-directed mutagenesis to introduce cysteine residues to different positions on the sugar-oxidizing enzyme cellobiose dehydrogenase, which contains an FAD-dependent dehydrogenase subunit and a heme-containing subunit. Both these subunits are naturally tethered by a flexible peptide linker such that the heme subunit can act as an electron transfer partner to the FAD-dehydrogenase subunit.^101^ By strategically placing individual cysteine residues for DET investigation, the authors provided convincing evidence that DET takes place exclusively through the heme-containing subunit (the FAD-dehydrogenase does not undergo DET).

The above-mentioned approaches are still limited as they utilize the functionality of the 20 “standard” proteogenic amino acids found in the structure of enzymes/proteins. An emerging approach (genetic code expansion, GCE) allows incorporation of unnatural amino acids (UAAs) into any site of a protein sequence, and thus permits the introduction of functional groups that are not naturally found in the enzymes/proteins. GCE exploits orthogonal tRNA/tRNA synthetase pairs to allow the site-specific incorporation of UAAs by decoding the amber (UAG) stop codon (termed amber codon suppression).^102−104^ The site-specific incorporation of UAAs can be used to improve enzymatic stability and catalytic properties, and to understand electron transfer mechanisms of metalloproteins/metalloenzymes.^105,106^ In one example, Schlesinger et al. targeted the orientation of the copper oxidase (CueO) on the electrode surfaces for improved DET, where the alkyne-containing UAA propargyl-l-lysine (PrK) was placed in different positions on the protein’s surface.^107^ One site was near to the type 1 Cu site and the other two were close to the trinuclear cluster (TNC) Cu site (a control was prepared with PrK located far away from both active sites). Site-specific immobilization of the mutants was performed by the Cu(I)-mediated cycloaddition (CuAAC) “click” reaction on GC electrodes functionalized with different azide-containing pyrene linkers. All of the mutants placing PrK in proximity to either the T1 Cu or the TNC exhibited improved *k*_ET_ with respect to their targeted electron transfer partner, demonstrating that this strategy was indeed effective at improving heterogeneous electron transfer. Further, all three of these mutants displayed improved electrocatalytic performance for O_2_ reduction by the enzyme.

The same PrK UAA was introduced to the cytochrome *c* unit that had been artificially fused to glucose dehydrogenase, which was subsequently immobilized on functionalized GC electrodes by click chemistry. A mutation close to the electron-transferring heme domain provided the largest current density on the electrode when compared to the introduction of PrK in proximity to the FAD cofactor of this chimeric enzyme. These site-specific mutations permitted the electron transfer properties of the FAD cofactor and the artificial heme domain to be investigated.^108^

An alternative UAA was used to improve the DET properties of a “small” laccase multicopper oxidase, where five positions on the surface of the enzyme were targeted for the introduction of an azide-carrying phenylalanine UAA (*para*-azidophenylalanine, pAzF). Three of the mutation sites were near to the TNC, one addition site was in proximity to the T1 Cu center, and the last position served as a control (in proximity to neither the TNC nor the T1 Cu site). Cyclooctynyloxyethyl-1-pyrenebutyrate (PBCO)-functionalized indium–tin oxide (ITO) electrodes were used for the Cu-free ring-strained “click” immobilization of the enzyme. Contrary to expectations, the mutant prepared as a negative control showed the greatest *k*_ET_. Interestingly, the structural analysis of laccase revealed the presence a water channel close to the basal site connected with the trinuclear copper cluster. Therefore, the immobilization of laccase from this basal site was attributed to this enhanced *k*_ET_, and resulted in stable current up to 8 days.^109^

Although these studies have demonstrated improved DET efficiencies, challenges still remain for the wider deployment of GCE in electrochemistry, such as relatively diminished expression yield (due to protein truncation during RNA translation), the cost of UAAs (when commercially available), and the unavailability of 3D structural information for some proteins/enzymes.^110,111^ However, recent advances to improve UAA incorporation and protein yields are promising developments in this area.^112,113^

#### 2.4.2 Bacteria Engineering for Enhanced Extracellular Electron Transfer

In recent years, the possibility to utilize synthetic biology to engineer intact bacteria, introducing non-native properties including the EET pathways of *Shewanella* or *Geobacter* in other organisms, has attracted particular attention and enabled enthralling research directions. Specifically, the approach is based on engineering the microorganisms with outer membrane cytochromes, thus overcoming the limitation of insulating membrane layers. The first reports of heterologous gene expression were reported for the Mtr pathway of *S. oneidensis* in *Escherichia coli* (*E. coli*), with the 2010 work of Jensen et al.^114^ While the engineered strain enabled a 4-fold faster reduction of insoluble Fe(III) compared to the wild-type *E. coli*, such reduction was still significantly slower compared to wild-type *S. oneidensis* MR-1, mostly due to the slow rate of the first electron transfer step to reduce MtrA. To tackle this aspect, following works have focused on the expression of cytochrome *c* CymA as an electron donor to the MtrA,^115^ as well as the role of exogenous flavins, to further boost electron transfer.^116^ Recently, Mouhib et al. showed that the complete expression of the Mtr pathway together with periplasmic cytochromes can further enhance electron transfer in engineered *E. coli*.^117^ Conversely from the expression of *Shewanella* EET pathways, considerably less studies investigated the expression of the *G. sulfurreducens* EET mechanism due to its higher complexity. Notably, Sekar et al. reported the expression of *G. sulfurreducens* outer membrane cytochrome OmcS in cyanobacteria, enabling a 9-fold current increase.^118^ Such heterologous expression was recently achieved also by Dong et al. in a previously engineered cyanobacterium for accomplishing NH_3_ production in a bioelectrochemical system without the need of exogenous diffusible redox mediators.^33^ Specifically, the engineered bacteria with OmcS allowed a 13-fold higher NH_3_ production compared to the strain not engineered with the heterologous EET pathway, together with an increased faradaic efficiency of about 23%. We refer the reader to recent reviews on the use of synthetic biology for microbial electrochemical systems for further insights into this topic.^119,120^

## 3 Methods to Study Enzymatic and Microbial Electrodes

### 3.1 Electroanalytical Methods

Protein film voltammetry (PFV) (also known as protein film electrochemistry) is a popular technique to study redox proteins, as the protein or enzyme is wired to an electrode and can be studied via variety of electroanalytical techniques. In PFV, the energy (or driving force) supplied to the enzyme is controlled via the applied potential, while the current provides kinetic and mechanistic information about the enzyme. PFV is attractive as only a small quantity of enzyme/protein is needed, the complex dependence of enzyme diffusion on the electrochemical signal can be eliminated, and a large range of conditions can be tested using a single bioelectrode, including those where the enzyme is only transiently stable (e.g., extreme pH).

**Figure 3:**
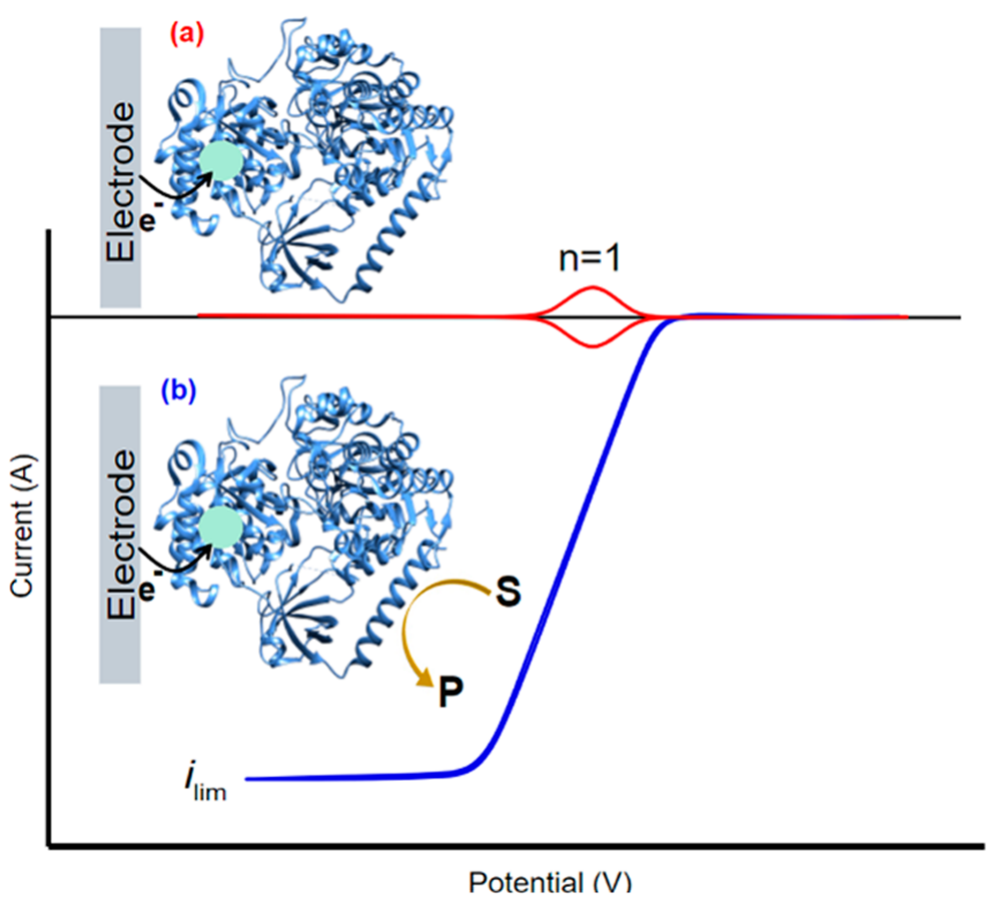
Idealized cyclic voltammetry showing the redox response of an enzyme under reductive conditions with one active redox center (*n* = 1) under (a) non-turnover conditions where there is only an electron transfer between the redox center of the enzyme and the electrode, and (b) turnover conditions in the presence of a substrate and steady-state conditions. The current is a direct measure of enzyme activity.

During PFV, electrons are transferred between redox centers of the protein/enzyme and the electrode surface (Figure 3a). Page et al. reviewed 31 redox proteins/enzymes and concluded that most redox cofactors undergoing physiologically relevant electron transfer reactions are found within 14 Å of each other.^121^ Marcus theory was subsequently adapted to enable *k*_ET_ to be predicted for redox proteins with knowledge of driving force and distances between redox cofactors, as well as the nature of the amino acid/solvent environment between cofactors (which is expected to impact their electronic overlap, *H*_DA_). Nevertheless, some important points must be kept in-mind: (i) electron transfer does not abruptly stop for distances >14 Å; (ii) X-ray crystal structures do not provide information on dynamic conformational changes of proteins (which could impact *k*_ET_); (iii) proteins have been known to deviate from Marcus behavior at electrode surfaces.^38^ Nevertheless, it remains intuitive that the redox cofactors of enzymes/proteins should be oriented for favorable electron transfer.

As discussed above, redox mediators are often used to overcome sluggish electron transfer in the case of DET.^122^ This is useful for performance driven applications such as those in biofuel cells, however extracting information solely about the enzyme is challenging as electrochemical responses of the mediator and enzyme are often convoluted.^123^ DET simplifies the interpretation of electrochemical signals and allows thermodynamic and kinetic parameters to be extracted with reduced inference.

Electroanalytical methods are also a unique tool to study microbial electrodes. The concepts presented for PFV applies also for the case of intact bacterial cells deposited on electrode surfaces, while keeping in mind the constrains introduced by the presence of the bacterial membranes previously introduced. The electrochemical characterization of microbial electrodes allows elucidating the role of redox intermediates in E-DET, as well as unveiling the presence of endogenous diffusible redox mediators produced by the bacterial cells on the electrode. Furthermore, the development of microbial biofilms on the electrode can also be studied by means of long-term potentiostatic polarization. The following sections present the various electrochemical methods employed for PFV and microbial electrodes characterization.

#### 3.1.1 Cyclic Voltammetry

Cyclic voltammetry (CV) is one of the most prevalent electroanalytical techniques employed for both PFV and microbial electrodes, as it is simple to use and the voltammogram provides information in a clear and visual manner. To perform CV, the potential of the working electrode is swept linearly back and forth between two potentials (*E* in volts (V)) (a single sweep is known as linear sweep voltammetry (LSV)), at a defined scan rate (ν in V s^–1^) and the current (*i* in amperes (A)) is recorded.^124^ For PFV under noncatalytic conditions, thermodynamic information about the redox centers of the protein/enzyme can be extracted if the scan rate is sufficiently low that the protein/enzyme remains under equilibrium conditions. Each peak in a non-turnover CV corresponds to a redox site undergoing oxidation/reduction. The total number of peaks (n) therefore equates to the total number of active redox sites in the protein/enzyme (Figure 2, red). Importantly, the positions of the reduction and oxidation peaks yield *E*^0^′, the widths of the peaks at half-height (Δ*E*_p1/2_) depends on the number of electrons (*n*) involved in that electron transfer process (i.e., *n* = 1, 2, etc.), and the peak current (*i*_p_) is proportional to the quantity of adsorbed redox species.^125^ One of the limiting factors of PFV under DET is the low enzyme surface coverage and unfavorable orientations of the enzyme on the electrode that can greatly reduce electron transfer efficiency. As a result, the response of the enzyme can become convoluted with capacitive charging at the electrode making interpretation of electrochemical signals challenging under non-turnover conditions. To overcome this, graphite is commonly used for PFV under DET conditions as its preparation is facile, the rough surface increases the contact points between the enzyme and electrode, and the oxide rich functionality provides an excellent environment for the electrostatic adsorption of proteins.^126^ Armstrong and co-workers demonstrated that the immobilization of fumarate reductase on pyrolytic edge plane graphite (PGE) was facile and allowed the enzyme to retain in an extremely electroactive state while maintaining its native structure.^127^ For this reason detailed kinetic studies have also been performed using PGE.^128^ Another approach to amplify enzymatic response under non-turnover conditions is Fourier-transformed AC voltammetry (FtacV). A large-amplitude sinusoidal wave is applied to the linear potential sweep during CV, after which the current is Fourier transformed to a power spectrum which allows the separation of faradaic and non-faradaic contributions.^129^ FtacV is a relatively new technique in PFV; however, over the past 5 years, Parkin and co-workers have been able to elucidate mechanisms of electron transfer in the Mo-containing YedY catalytic subunit,^130^ the HypD [NiFe]-hydrogenase maturase protein,^131^ as well as separating the catalytic and electron transfer current contributions in [NiFe] hydrogenase.^132^

Altering the scan rate under non-turnover conditions provides kinetic information about the protein/enzyme. Fast scan rates have been able to access coupled electron transfer processes between the protein and electrode and the associated rate constant (*k*^0^). Hirst et al. demonstrated scan rates up to 3000 V s^–1^ could be used to probe the electron-exchange characteristics of azurin,^133^ a copper containing redox protein, where a k^0^ was determined to be approximately 5000 s^–1^. Using scan rates up to 3000 V s^–1^ also allowed coupled electron transfer processes occurring for three different ferredoxin proteins in the millisecond time domain to be resolved.

In the presence of an enzyme’s substrate, the application of a sufficient driving force for electron transfer (*E*_applied_) can result in “catalytic” currents. In this case, an enzyme that has been oxidized by an electrode can be reduced by the oxidation of its substrate, leading to a second round of oxidation (of the enzyme) by the electrode. The magnitude of this catalytic current is dependent on *E*_applied_, as well as the product of the enzyme’s turnover frequency and the quantity of enzyme on the electrode surface (strictly, the quantity of enzyme undergoing DET). This second factor is not always easily determined. Further, the confinement of an enzyme to an electrode enables the simple titration of substates within the electrochemical cell; this, in turn, enables the determination of apparent kinetic parameters such as Michaelis constants (*K*_M_). If only one cofactor is in communication with the electrode, the formal onset potential for catalysis can be loosely related to the formal potential for substrate reduction (for reduction *E*^0^′_onset_ ≈ *E*^0^′_red_), provided the scan rate is sufficiently low that the protein remains at equilibrium. Parameters such as temperature, pH and ionic strength should also be considered as they can also significantly perturb this value. The appropriate potential window should also be chosen to avoid overpotential deactivation of the enzyme. For slower scan rates where the enzyme is maintained in a state of equilibrium a reversible CV may be obtained (Figure 3a, blue).^124^ Conversely, peak separation or hysteresis observed at elevated scan rates can be indicative of slow reaction kinetics or protein instability/desorption from the electrode.

Hydrodynamic electrochemistry is often used to study enzyme kinetics as mass transport limitations can be removed. The electrode is rotated to induce a controlled flux of fluid to the electrode surface by laminar flow, where the substrate is continually replenished. When a steady-state regime is reached, the rate-limiting step of the system becomes enzyme-dependent (Figure 3, blue line) enabling the investigation of various processes such as intramolecular electron transfer, conformational changes, ion-ligand exchange, or the binding/release of substrates/products within the enzyme.^134^

For the specific case of microbial electrodes, CV are particularly useful to investigate the mechanism of EET. Specifically, performing CV under noncatalytic and catalytic conditions allows one to study the presence of membrane bound redox proteins responsible for E-DET, as well as the presence of endogenous diffusible mediators responsible for E-MET. The two EET modes can be distinguished by first acclimating the microbial electrode to a specific electrolyte prior to gently washing and transferring the electrode to a fresh electrolyte and immediately performing additional CVs. If the EET is taking place primarily through endogenous diffusible mediators, the CV under turnover should reveal a loss of catalytic response in the new electrolyte as shown by Marsili et al. for the case of endogenous riboflavin produced by *S. oneidensis*.^67^ Conversely if E-DET is taking place, the catalytic response should be (at least partially) retained. In the latter case, performing CV under non-turnover conditions would further allow studying the redox potentials and eventually characterizing the involved redox centers responsible for E-DET.

Modeling of experimentally obtained CV can be performed to gain critical insights into the EET process,^135^ such as unveiling microbial electrodes with dual electron transfer mechanisms (both direct and mediated),^136^ or studying the rate-determining step in the EET process (i.e., for a microbial anode: (i) mass transport of the substrate in the microbial biofilm formed on the electrode, (ii) microbial turnover of the substrate, (iii) reduction of the redox intermediate, (iv) transfer of electrons throughout the biofilm, and (v) electron transfer at the electrode surface).^137^

Since several molecules and enzymes can be secreted by bacterial cells into the electrolyte, when E-MET is revealed, the electrolyte can be filtered utilizing membranes with different nominal molecular weight cutoff to gain details on the origin of the redox response. Specifically, performing CV with the filtered electrolytes allows one to estimate the range of molecular weight of the endogenous redox molecules and/or secreted enzymes responsible for the redox response, as recently shown by Saper et al.^68^ Finally, an extremely interesting aspect of microbial electrodes is that their composition might vary based on the conditions under which the electrodes were formed and/or exposed. If a microbial electrode is formed under electrochemical polarization, CVs could be used to characterize the effect of the utilized potential on the catalytic response,^138^ revealing for example different onset potentials for the catalytic reaction. This aspect is particularly important for microbial electrodes since bacterial species can over/under express specific genes depending on environmental conditions and external stress. As a general example, it has been reported that *G. sulfurreducens* can express more than 100 types of cytochromes,^139^ showing an outstanding flexibility on EET pathways depending on the local environment and physicochemical conditions, which could be characterized based on different CV responses.

#### 3.1.2 Amperometry and Chronoamperometry

Amperometry is an electrochemical technique which measures *i* as a function of time (*t*) at a constant *E*_applied_. For the purposes of this tutorial review, we define chronoamperometry (CA) as a technique involving at least one step in applied potential (*E*_applied_), whereas amperometric *i*–*t* curves (or constant potential chronoamperometry) refers to the measure of *i* vs *t* at a single potential. We also acknowledge that CA generally considers experiments in which an analyte/substrate is not added over time (unlike in the case of amperometric *i*–*t*).

Amperometry is widely used in biosensors development for the quantitative detection of analytes, starting from the first glucose biosensor developed in 1962 by Clark.^140^ Today, modern glucose biosensors constitute a large proportion of commercially available amperometric biosensors,^141^ as well as numerous others employed in the environmental monitoring of hazardous compounds such as peroxides and aromatics,^142,143^ explosives like TNT,^144^ and harmful pesticides.^145,146^ With microbial electrodes, amperometric *i*–*t* curves allowed determining the influence on the current response of heavy metals,^31,32,147^ pesticides,^148^ organic carbon load^149^ and other toxic compounds,^150^ obtaining microbial biosensors for various applications. Amperometry is extensively utilized also for the characterization of biofuel cells and biosupercapacitors,^141,151^ an important area of research as investment for renewable energies continues to grow. Furthermore, amperometric *i*–*t* curves are particularly useful to study biophotocurrent generation during artificial light/dark cycles with photosynthetic apparatus/enzymes^152,153^ and photosynthetic bacteria^73,81,154,155^ In this case, the amperometric test is performed while alternating light/dark cycles by means of an artificial light source, and can provide insights into the kinetic of the photoinduced electron transfer reaction between the biophotocatalysts and the electrode.

CA is often chosen for fundamental enzymatic research as kinetic and mechanistic information can be obtained. During CA the applied potential is stepped and the current is recorded. This is particularly helpful since the turnover rate of the biological catalyst can be precisely controlled via the applied potential. Furthermore, the current is a direct measure of enzyme turnover, any changes in current magnitude show how the activity of the enzyme is changing in real time.^156^ Under non-turnover conditions, CA also permits heterogeneous *k*_ET_ to be determined for surface-confined bioelectrodes.

Amperometric *i*–*t* curves can be used in combination with a rotating disk electrode (RDE) to determine the apparent *K*_M_ of a redox enzyme, an important kinetic parameter to describe the affinity between an enzyme and substrate. The *K*_M_ value can be easily determined by recording the enzyme activity as a function of substrate concentration and fitting data to the Michaelis–Menten equation. This approach was nicely demonstrated by Léger and co-workers where the *K*_M_ of periplasmic nitrate reductase from *Rhodobacter sphaeroides* was determined during nitrate reduction.^157^ Under the steady-state regime, a constant and large overpotential was applied to drive nitrate reduction, while the substrate concentration in the cell was increased stepwise. The entire experiment lasted only 150 s, and the *K*_M_ value could be determined directly from the stepwise current profile. This approach is also useful for less stable protein films as the experiment can be completed relatively quickly. Similarly, amperometric *i*–*t* curves have been performed with RDE for microbial electrodes, studying mass transport inside the microbial biofilms obtained on electrode surfaces.^158^ Performing photoamperometric *i*–*t* curves with RDE in the presence of *Rhodobacter sphaeroides* cells and dichlorobenzoquinone as an exogenous diffusible mediator at different concentrations, Kasuno et al. showed that the biophotocurrent production from intact bacteria-based photoanodes follows a Michaelis–Menten-type kinetic model.^159^

Amperometric *i*–*t* curves are also valuable for studying inhibition kinetics under steady-state conditions, as active and inactive states of the biocatalyst can be accessed at different potentials. This is particularly useful for investigating the aerobic inhibition of redox enzymes, such as hydrogenases. The rapid denaturation of these enzymes under O_2_ presents a major challenge for their implementation into biotechnologies making the understanding of their O_2_ inhibition mechanisms of critical importance. An additional issue for hydrogenases is that those with the efficient H_2_ turnover are also particularly oxygen sensitive. Using PFV, Léger et al. investigated the permanent deactivation and reversible inhibition of *Cr*HydA1, a [FeFe]-hydrogenase isolated from *Chlamydomonas reinhardtii* with CO and O_2_ during H_2_ oxidation.^160^ Rate constants (*k*_i_) for reversible inhibition with CO and deactivation with O_2_ were determined by altering the gas composition in the headspace of the cell, and observing relative changes in current from the amperometric trace. The results can be utilized also to determine the reaction order by plotting *k*_i_ as a function of inhibitor concentration.

More recently, the reversible inhibition kinetics of [FeFe]-hydrogenase with hydrogen sulfide (H_2_S) have been studied by Léger and co-workers. Inhibition by sulfide renders the enzyme inactive and protected from O_2_.^161^ The electrode was stepped between two potentials (predetermined via CV) where (i) inactivation with sulfide was known to occur and (ii) the enzyme was fully active. The current response immediately following the potential step was fitted to kinetic model and used to determine rate constants of inhibition and reactivation.

#### 3.1.3 Square Wave Voltammetry

**Figure 4:**
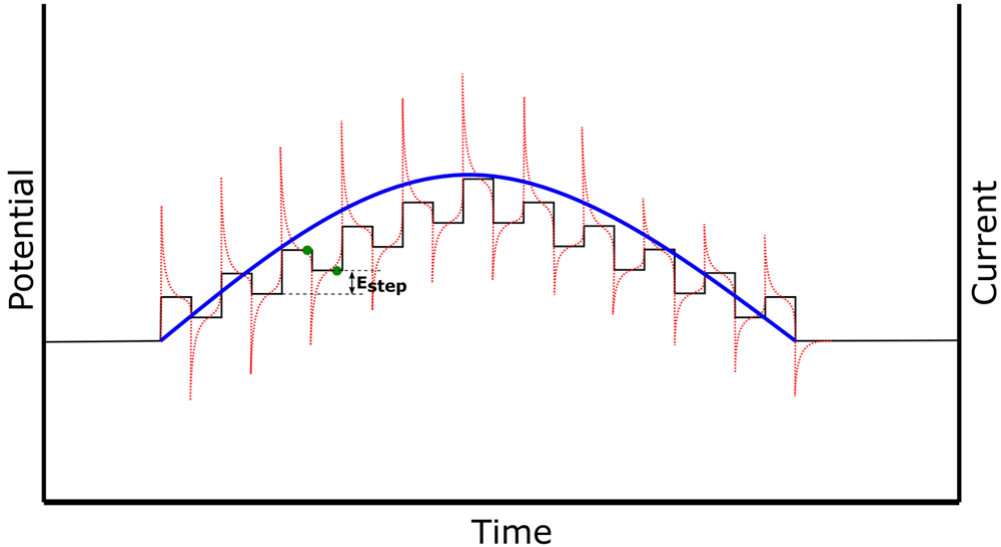
SWV trace where the potential (black) is applied as a staircase potential, the absolute current (red) is sampled twice at the end of each potential step (green dots), and the final current is reported as an average of both values (blue line).

Square wave voltammetry (SWV) is among the fastest and most sensitive pulsed electroanalytical techniques, as it allows diminishing the non-faradaic contributions to the current response. This paves the way to the detection of chemical species at nanomolar concentrations, establishing the operating principle behind many electrochemical sensors.^162−166^ In SWV, the potential is swept linearly as a modulated staircase with a defined step height (Figure 4).

The non-faradaic contribution is mitigated as the current is sampled twice (at the end of each potential step) and reported as a difference of the two values. SWV exploits the relative decay rates of faradic and non-faradic currents as the contribution of capacitive charging is negligible by the time the current is sampled at the end of each potential pulse.^162^ This discrimination is also useful for when using nanostructured electrodes in PFV, as a large surface area leads to significant capacitive currents, and removes the need for baseline subtraction which may not be accurate. This allows for a more accurate calculation of the electroactive enzymatic surface coverage on an electrode and the value of *k*_ET_. Leopold and co-workers demonstrated that SWV can be utilized for determination of the *k*_ET_ of the azurin redox protein monolayer on different alkanethiol SAMs (on a gold nanoparticle).^167^ The values of Δ*E*_p_ during SWV were used to determine *k*_ET_ by Reeves and co-workers.^168^ SWV can also be used to assess the activity of certain surface confined proteins. Sun et al. demonstrated SWV could be used in combination with Co(bpy)_3_ as an electroactive indictor to investigate the antioxidant activities of 4 different flavonoids which were used to probe the oxidative damage of proteins at a graphene-based electrode (protections of bovine serum albumin protein from damage on functionalized graphene-based electrodes by flavonoids).^169^ The activity of each flavonoid protein could be assessed simply by comparing the relative peak intensity of each SWV peak. In addition, SWV is also a valuable technique in protein monolayer electrochemistry which is used to study the electron transfer properties of immobilized proteins (the reader is directed to a review by Campbell-Rance et al.^167^).

The application of SWV in PFV remains reasonably limited due to the complex nature of the potential modulation, however it has been utilized for the operation of biosensors due to its high sensitivity.^170,171^ Recently, Monteiro et al. constructed a disposable biosensor with multiheme cytochrome *c* nitrate reductase on pencil graphite for the detection of environmental cyanide,^172^ where SWV was used to determine the sensitivity and response of the bioelectrode under turnover conditions. SWV is also valuable in the case of nanostructured electrodes with a high capacitance. Applications of SWV in PFV for kinetic studies still remains fairly limited, however a number of theoretical studies have modeled the electrochemical signals which may be expected to aid future experimental work.^170,173,174^ The application of SWV for the characterization of microbial electrodes is also not common, however, the technique provides a powerful tool for the study of the redox intermediates at play in the EET process. Yates et al. utilized SWV to accurately identify the midpoint potential for electron uptake in *Marinobacter*–Chromatiaceae–*Labrenzia*-based biocathodes performing oxygen reduction, with the aim to study the long-distance EET transfer process in an electroautotrophic microbial community.^175^

### 3.2 Hyphenated Electrochemical Techniques

#### 3.2.1 Spectroelectrochemistry

Spectroelectrochemistry is a hyphenated technique that combines two classical methods, electrochemistry and spectroscopy, to obtain chemical information.^125,176,177^ Electrochemistry can be coupled with different spectroscopic methods, mainly with absorption spectroscopy in the UV–vis,^178^ IR,^179^ X-ray range, as well as Raman scattering spectroscopy,^180^ and electron paramagnetic resonance.^181^ Other techniques, such as nuclear magnetic resonance (NMR),^182^ X-ray absorption spectroscopy (XAS),^183^ and luminescence^184^ can be used with electrochemistry but are less common.^185^ There are several applications for spectroelectrochemical techniques in organic and inorganic chemistry, biochemistry, materials, and nanomaterial science. We focus this section mainly on UV–visible spectroelectrochemistry and its use to investigate proteins/enzymes, and fluorescence spectroelectrochemistry for the study of EET in microbial electrodes.

A few challenges are faced when performing spectroelectrochemical experiments for bioelectrochemical systems, resulting in their limited implementation. Specifically, the experimental setup needs to be designed taking into consideration the light source (in UV/vis and fluorescence spectroscopy), introducing various restrictions such as the use of grid or transparent electrodes. León et al. described in detail the design of electrochemical cells for different spectroelectrochemical analysis.^176^ While electrochemical analysis of redox proteins/enzymes can be performed with limited quantities (less that μg), this is not always the case with spectroelectrochemistry, since sufficient sample should be provided to be analyzed by the two techniques simultaneously. To this end, a monolayer of proteins adsorbed on an electrode surface often does not provide sufficient signal for the secondary technique; with an example being a redox-dependent absorption with a relatively low molar extinction coefficient (ε). Since an increase in sample concentration should not be possible for the case of electrodes with a monolayer coverage of protein, a possible approach to increase the absorbance signal, considering Beer–Lambert law, is to increase the measurement path length, which could be achieved using proteins in solution (not adsorbed on an electrode). However, this is not compatible with efficient DET and it is often necessary to use MET. Among the drawbacks of the approach we can mention the following: (i) a bulk (at least localized) electrolyte is required, which increases the time required to reach equilibrium; (ii) MET requires either that the mediator oxidation/reduction does not interfere with the feature of interest (or background subtraction would be required), or that the sample is followed at an isosbestic point of the mediator (which is often not at the λ_max_ of the feature of interest).

**Figure 5:**
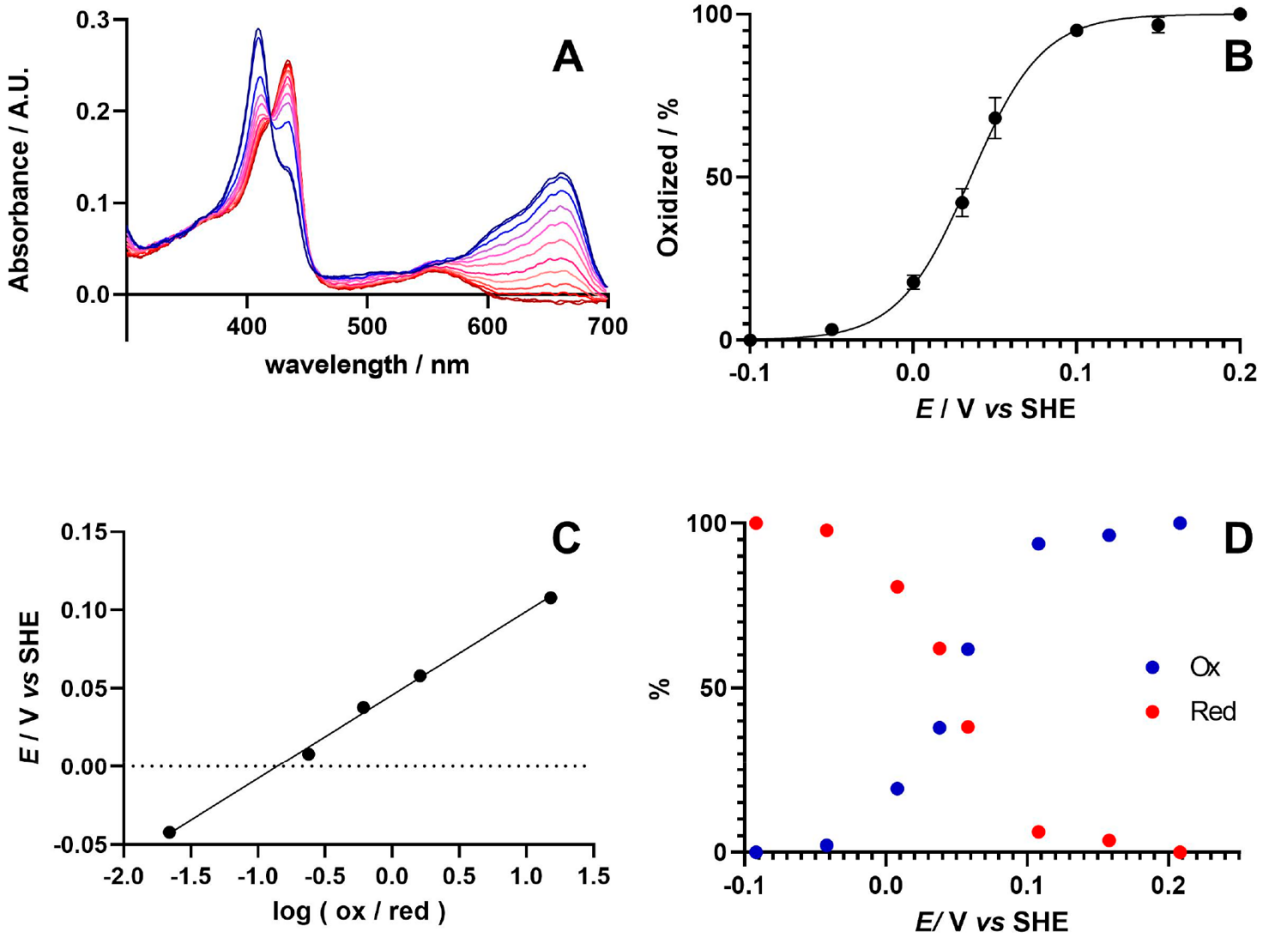
(A) Spectroelectrochemical oxidation (blue) and reduction (red) of myoglobin in the presence of methylene blue as an electron mediator. (B) Nonlinear regression and (C) linear fit of data obtained in (A) to the Nernst equation to determine the formal redox potential (*E*^0^′) and the number of electrons transferred (*n*). (D) Changes in the oxidized and reduced features of myoglobin’s heme cofactor at different applied potentials. Adapted with permission under a Creative Commons Attribution License from ref (186). Copyright 2022 The Authors.

Spectroelectrochemistry is considered an in situ technique, meaning that it is possible to simultaneously realize spectroscopic measurement within an electrochemical experiment.^176^ In Figure 5, we give an example of a spectroelectrochemical experiment performed in the Milton group to evaluate the *E*^0^′ of the heme-containing protein myoglobin.^186^ In the study by Guo et al., myoglobin proteins were reconstituted with various artificial cofactors (different metals and different porphyrin ligands) to enhance their natural peroxidase activity. It was proposed that a change in *E*^0^′ of the redox cofactor could explain the observed improved activity of one of these myoglobin variants; indeed, spectroelectrochemistry permitted the *E*^0^′ of the wild-type and a variant myoglobin to be determined in solution and confirmed a significant increase in *E*^0^′ for myoglobin containing the artificial cofactor. The spectroelectrochemical cell contained a Pt grid electrode as the working electrode, a Pt wire as counter electrode (shielded behind a porous-glass junction), and a Ag|AgCl (3 M KCl) reference electrode. A reduction potential titration was performed by stepping the potential from 0.0 to −0.3 V at 50 mV intervals. Each step lasted 1200 s to ensure equilibration of the ox/red states of myoglobin. For each step, an average of absorbance values recorded in the last 30 s was taken (Figure 5A). The Soret band of oxidized myoglobin is observed at 410 nm (ε_410_ = 157 mM^–1^ cm^–1^) in the oxidized form, which shifts to 434 nm in the reduced form.^187^ Using the Beer–Lambert law, it is then possible to evaluate the ratio between the [Ox] and [Red] form at each applied potential. A graph of absorbance vs *E* is sigmoidal and can be fit by nonlinear regression to the Nernst equation (Figure 5B):
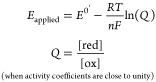
Thus, fitting the Nernst equation also enables the value of *n* to be determined for each redox feature (Figure 5C,D).

In one example, Wohlschlager et al. used UV/vis spectroelectrochemistry to generate the activating radical species for glyoxal oxidase (GLOX, an extracellular source of H_2_O_2_ in white-rot secretomes, where it acts in concert with peroxidases to degrade lignin), to continuously measure its concentration, and to simultaneously measure the catalytic activity of GLOX based on its O_2_ consumption.^178^ Spectroelectrochemical measurements were carried out in a 3 mL quartz cuvette optically transparent electrochemical cell comprising a three-electrode setup with a machined glassy carbon tile as the working electrode. The electrochemical generation of ABTS^+^ was controlled by applying electrochemical pulses of 600–700 mV vs Ag|AgCl for 1–10 s (depending on the desired quantity of ABTS^+^ to be generated, followed by spectroelectrochemistry at 420 nm), while monitoring O_2_ concentrations using an oxygen microsensor. Chen et al. utilized UV–vis spectroelectrochemistry to investigate riboflavin (RF), the primary redox-active component of flavin cofactors which is involved in many redox processes in biogeochemical systems.^184^ While the redox behavior and reaction mechanisms of RF in hydrophobic sites remain unclear, it is known that it possesses three accessible oxidation states (similar to quinones), each with protonated and deprotonated forms. In the oxidized form, the flavin shows two peaks at ≈360 and ≈450 nm.^188^ In their study, Chen et al. integrated spectroelectrochemical analysis and density functional theory calculations to explore the redox behaviors of RF in dimethyl sulfoxide (which was used to create a hydrophobic environment). In situ UV–vis spectra of RF at different *E*_applied_ were obtained to spectroscopically observe the relative change in species during reduction. The absorption peaks at 446, 344, and 271 nm were observed to decrease with decreasing potential (more negative values), along with an increase of absorption peaks at 330 and 262 nm (corresponding to the reduction of RF). Absorption at 373 nm dramatically increased, to a following decrease after the applied potential reached values more negative than −1.0 V vs Ag wire. The slight change in intensity of the two absorption peaks, 373 and 475 nm, indicates that the radicals formed during the reduction were long-lived. A different approach was used by Roy et al. where UV–vis spectroelectrochemistry was used to study a molecular cobalt phthalocyanine catalyst that reduces CO_2_ to CO. This catalyst has four phosphoric acid functional groups that can be used as anchoring groups to immobilize the catalyst on metal oxide electrodes.^189^ The reduction of CoPcP/[CoPcP]^−^/[CoPcP]^2–^ was performed by applying different potentials from 0.21 V to −0.99 V vs SHE while simultaneously recording UV–vis absorbance, revealing a potential-dependent change in normalized absorbance. The feature at 626 nm (corresponding to CoPcP) disappeared, and two new features at 439 and 717 nm appeared, consistent with the formation of [CoPcP]^2–^. The stability of [CoPcP]^2–^ at more negative potentials was then evaluated under N_2_ and CO_2_ by recording the UV–vis spectrum. Under a N_2_ atmosphere, the spectrum remained unchanged, although in the presence of CO_2_ (for *E*_applied_ < −0.79 V vs SHE) the [CoPcP]^2–^ state was found to decrease (the two features at 439 and 717 nm decreased in intensity), consistent with the reduction of CO_2_ by the reduced [CoPcP]^2–^ state of the catalyst. Due to this spectroelectrochemical approach, they were able to study the stability of the catalyst in the presence or absence of CO_2_, and its behavior at different applied potentials. Finally, they integrated CoPcP to a mesoTiO_2_ system with a p-type silicon photoelectrode to achieve an improved turnover number with 66% selectivity for CO formation in aqueous conditions.

IR spectroscopy can be coupled with electrochemistry where ideally a change in potential (and the oxidation state of the species) affects the IR absorption spectrum, providing information about the identity of the elements (i.e., a ligand bound to a cofactor), and the structural composition of the molecule. It is also applied to enzymes such as hydrogenases and nitrogenases (and many more), metalloenzymes catalyzing H_2_ formation and N_2_ fixation, respectively. IR spectroelectrochemistry has been utilized for the study of [FeFe]-hydrogenase that contains an iron-based catalytic cofactor called the H-cluster, which is composed of two subclusters: a [4Fe-4S]_H_ and a binuclear cluster [2Fe]_H_. These subclusters are coupled with a cysteine residue, and the diiron cluster has cyanide, carbonyl, and hydride ligands. CO/CN^–^ stretching vibrations are specific for changes in the geometric and electronic configuration when redox and protonation reactions take place, making IR spectroelectrochemistry a valuable technique for the study of this system.^190^ Duan et al. studied [FeFe]-hydrogenase mutants in which the native aminodithiolate group of the [2Fe]_H_ was replaced with synthetic dithiolates.^191^ They were able to obtain a quantitative comparison of CO inhibition and reactivation kinetics for cofactor variants and to investigate the geometry of the H-cluster in the reduced form. They proposed an intrinsically flexible diiron site geometry that stabilizes polar ligands at the distal iron ion in catalytic intermediates and inhibited species. IR spectroelectrochemistry was also used to investigate the stability and the catalytic activity of [FeFe]-hydrogenase from *Clostridium beijerinckii* (CbHydA1) after exposure to oxygen.^179^ When exposed to oxygen, the H-cluster of the [FeFe]-hydrogenase was observed to form an inactivated state (*H*_inact_). IR spectroelectrochemistry revealed that the transition from *H*_ox_ to *H*_inact_ involves 1*e*^–^ and takes place at a relatively low potential (*E*^0^′_ox/inact_ = −0.38 V vs SHE at pH 8.4). Furthermore, *H*_inact_ could be reached by (electro)chemical oxidation in the absence of O_2_. Recently, the Vincent group demonstrated that this approach could be extended to study individual crystals of [FeFe]-hydrogenase from *Clostridium pasteurianum* (“CpI”), using a cocktail of electron mediators to shuttle electrons and ultimately control the redox state of the enzyme during FTIR microspectroscopy.^192^ Importantly, this approach allowed all of the catalytically relevant states of CpI to be probed, in addition to identifying a previously undetected catalytic state (*H*_redH_^+^).

IR spectroelectrochemistry has also been utilized to study Mo-dependent nitrogenase. Paengnakorn et al. employed a range of low-potential Eu complexes to mediate electron transfer to the MoFe protein and the coordination of CO to the FeMo cofactor active site of the MoFe protein was followed by in situ IR spectroscopy.^193^ Interestingly, the coordination of CO to the FeMo cofactor was demonstrated to be dependent on the potential of the solution (and the redox state of the protein), being triggered at *E*_applied_ more negative than −0.7 V vs SHE. This indicates that the FeMo cofactor requires more reduced levels in order to bind CO, although this reaction is known to take place when using the standard electron-donating Fe protein of nitrogenase (*E*^0^′ ≈ −0.43 V vs SHE).^194^

Fluorescence spectroelectrochemistry is gaining interest to shed light on the complex EET processes taking place in microbial electrodes. Specifically, the technique has been utilized to investigate the redox intermediates at play in the electron transfer and the interaction between exogenous redox mediators and the microorganisms due to the autofluorescence of various components of microbial cells, such as *c*-type cytochromes of *G. sulfurreducens* or photosystem II (PSII)-associated chlorophylls in cyanobacteria and algae. Schmidt et al. built a custom-made spectroelectrochemical reactor using transparent ITO electrodes to study the potential-dependent autofluorescence of *G. sulfurreducens* microbial biofilms.^195^ The in situ fluorescence emission spectra recorded in multistep spectroelectrochemical experiments allowed identifying the formal potentials of the cytochromes involved in the EET process, and a possible Förster resonance energy transfer (FRET) taking place for the reduced biofilm but not for the oxidized biofilm. Recently, Lemaître and co-workers combined electrochemistry and pulse amplitude modulation fluorescence to monitor photocurrent production from suspended algae (*Chlamydomonas reinhardtii*) in the presence of a diffusible redox mediator (2,6-dichloro-1,4-benzoquinone) while studying the non-photochemical quenching and photochemical PSII efficiency.^196^ To achieve this, the authors designed an electrochemical cell using a glass tube with an ITO-coated glass as the working electrode placed at the bottom of the tube. The fiber of a pulse–amplitude–modulation machine was then used to guide the lights used for excitation and fluorescence measurements, pointing at the bottom of the electrochemical cell. Amperometric *i*-*t* tests were performed at +0.9 V vs Ag|AgCl while measuring fluorescence under three different light conditions (i.e., dark, white actinic light, and saturating pulse) before and after the addition of the exogenous redox mediator. The developed method enabled the oxidation state of the mediator to be followed (by fluorescence for the oxidized form, and by electrochemistry for the reduced form), as a drop in biophotocurrent production (the photosynthetic apparatus of the algae decreasing the reduction rate of the mediator over time) was mirrored by an increase in non-photochemical quenching performed by the oxidized form of the exogenous quinone. Furthermore, the study revealed a complex evolution over time of the photochemical PSII efficiency. Over a short time, the addition of the exogenous mediator increased the photochemical PSII efficiency, possibly by rerouting “excess electrons” that could otherwise induce photosynthetic damage. However, over longer timeframes the photochemical PSII efficiency decreased, revealing a complex interplay between non-photochemical quenching and toxicity of the exogenous quinone mediator.

#### 3.2.2 Electrochemical Quartz Crystal Microbalance with Dissipation

**Figure 6:**
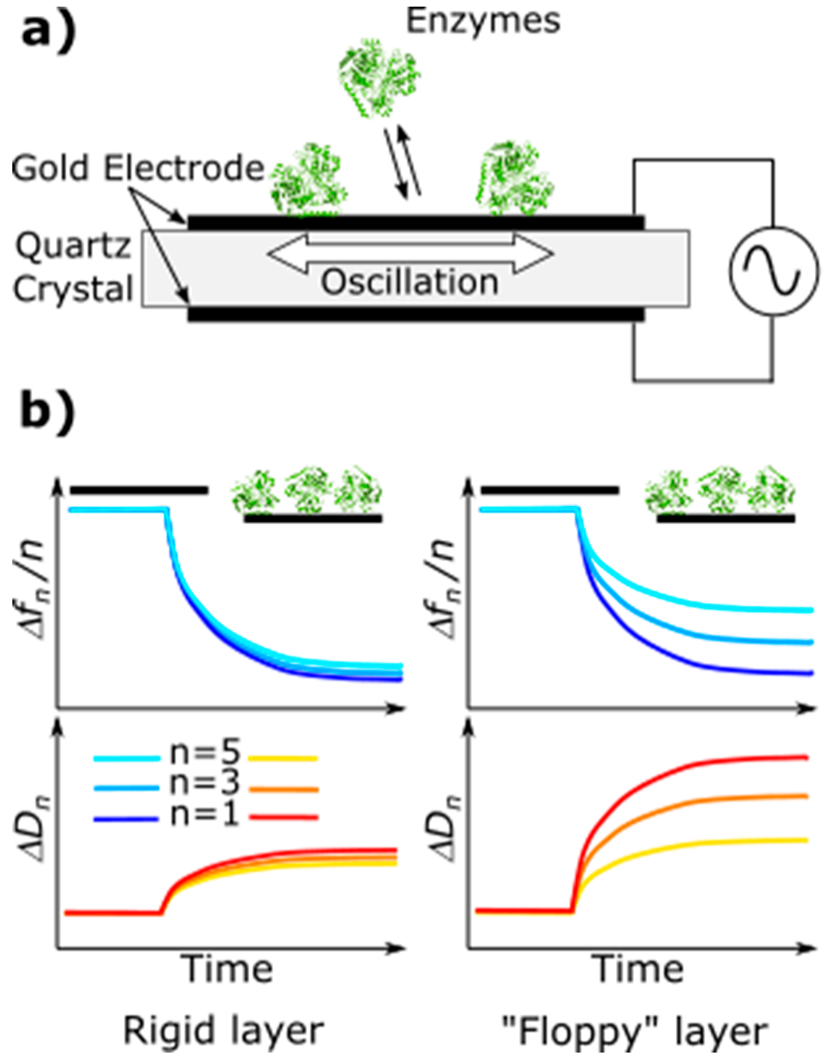
(a) Working principle of the QCM-D. The quartz disk is oscillated at its resonance frequencies with an alternating electric field. (b) During the adsorption of proteins on the surface, the resonance frequencies decrease (Δ*f*) and the dissipations increase (Δ*D*). Typical responses for a rigid and “floppy” add-layer are shown on the left and right, respectively.

Quartz crystal microbalance with dissipation monitoring (QCM-D) represents a robust surface-sensitive technique to monitor the adsorption of proteins. The sensor of a QCM-D device consists of a piezoelectric quartz disc. Alternating electric fields are applied between the faces of the disc, which is oscillated at its resonance frequencies (Figure 6a). A QCM-D instrument measures changes in the resonance frequencies (Δ*f*_*n*_) and in the dissipation signals (Δ*D*_*n*_) of the piezoelectric quartz crystal. Δ*D*_*n*_ values represent the changes of the energy dissipated by the adsorbed film for each resonance frequency and are determined from the characteristic decay time-constants of the corresponding oscillations. The lowest resonance frequency oscillation is called the fundamental mode (*n* = 1) and the higher frequencies are named overtones (*n* > 1). Since the changes in resonance frequencies are proportional to the overtone number, most of the instruments display normalized frequency changes Δ*f*_*n*_/*n*. When objects adsorb on the crystal surface, Δ*f*_*n*_/*n* and Δ*D*_*n*_ values increase and decrease respectively (Figure 6b). If the film is rigid, the normalized frequency changes can be directly related to the adsorbed amount using the Sauerbrey equation.^197^ A typical response for a rigid film is characterized by the same Δ*f*_*n*_/*n* values and little or no changes in Δ*D*_*n*_ (Figure 6b, left). When the adsorbed layer is “floppy” and dissipative, a split in Δ*f*_*n*_/*n* and Δ*D*_*n*_ signals can be observed (Figure 6b, right). The collection of Δ*f*_*n*_ and Δ*D*_*n*_ for the different resonance frequencies allows for quantitative information on the mass, thickness, and viscoelastic properties of the adsorbed layer.^198,199^

Further, electrochemical and QCM-D measurements can be performed simultaneously permitting correlation between film structures and electrochemical properties. This technique is commonly referred as electrochemical quartz crystal microbalance E-QCM-D. In the case of electroenzymatic catalysis, comparison of the amount of adsorbed enzyme, as measured with QCM-D, with electroenzymatic activity can give insight on the optimal orientation attached enzyme should have to maximize the electron transfer.^200−202^

Azurin has been found to irreversibly adsorb on a gold electrode modified with a self-assembled monolayer of octanethiol.^201^ The maximum surface concentration was independent of the protein solution concentration; for all the concentrations studied, the maximum adsorbed amount was 25 ± 1 pmol cm^–2^, closely corresponding to a monolayer coverage. Only small changes were detected in the dissipation signal suggesting a rigid layer structure. CV measurements performed on the same surface have shown electrochemical activity consistent with a one-electron, surface-confined Cu^2+/1+^ azurin redox couple. Using the peak area of the reduction peak, the surface coverage of electrochemically active azurin was estimated to be 7 ± 1 pmol cm^–2^. This discrepancy between QCM-D and cyclic voltammetry data suggested that not all the adsorbed proteins might have been electrochemically active.^201^ The adsorption of laccase on carboxyl- (Au-R^–^) and amine (Au-R^+^)-terminated ethylphenyl layers on gold electrodes has also been studied by the EQCM.^200^ Even if the amount of adsorbed enzyme was similar for both surfaces, the electrocatalytic current for oxygen reduction was significantly smaller when the proteins were adsorbed on the Au-R^–^ surface compared to the Au-R^+^ surface. Different orientations of the proteins on the electrode surfaces were hypothesized to be the cause of variation in electrocatalytic activity between Au-R^–^ and Au-R^+^ functionalization, likely due to the single-entry point of electrons via the “type 1” Cu of laccase (with one orientation favoring DET over the other). Furthermore, E-QCM-D can be used to optimize the enzyme deposition conditions to increase the electrocatalytic activity of the adsorbed species. It has been shown that maximum surface load does not always produce the best electroactive film, suggesting that knowledge of the enzyme-adsorbed amount is an important parameter to maximize the electrode efficiency.^203^ Bilirubin oxidase has been found to have maximum catalytic activity when adsorbed on unmodified and carboxylate-functionalized gold-coated sensors from a 15 mg mL^–1^ solution at pH 6.0. These optimal adsorption conditions could originate from a balance between rates of adsorption, reorientation, and unfolding for the protein adsorbed on the electrode surface.^203^

Hydrogenase (H_2_ase) and formate dehydrogenase (FDH) adsorption and electroactivity on a range of charged and neutral SAM-modified gold electrodes has been investigated using E-QCM-D.^202^ Using different types of SAMs, the effects of electrostatic and H-bonding interactions have been resolved, with electrostatic interactions having been found to be important for optimal enzyme orientation and electrocatalytic activity. Positively charged SAMs (SAM+) have shown near quantitative binding of H_2_ase and FDH in the correct orientation for direct electron transfer. In contrast, enzymes adsorbed on neutral and negatively charged SAMs have shown non-optimal orientation with reduced electroactivities. This behavior has been rationalized with the electrostatic attraction between the negatively charged region surrounding the distal FeS cluster of H_2_ase, or the FeS cluster of Fdh, and the SAM+. Using the enzyme loading obtained by E-QCM-D, the turnover frequencies (TOFs) of H_2_ase and FDH adsorbed on SAM+ have been found to be around 2 orders of magnitude smaller than the values found for the enzymes in solution. Since non-optimal orientation could be ruled out, the reason for these discrepancies is not clear, with one possible explanation being that not all the adsorbed enzymes are electroactive due to protein denaturization upon adsorption on the substrate. The stability of the adsorbed layers during electrochemical activity can also be followed using E-QCM-D. For H_2_ase adsorbed on different types of self-assembled monolayers, desorptive enzyme loss is found when H-bonding is not present at the enzyme–electrode interface.^202^ For bilirubin oxidase, the catalytic activity decreases even if the adsorbed mass remains stable. However, reduction in activity is associated with an increase in stiffness of the adsorbed layer, suggesting structural rearrangements as the primary mechanism of activity loss.^204^

One of the limitations of QCM-D consists in its capability on detecting what is called “wet” mass, which includes the adsorbate mass and the water coupled in the layer. Data analysis might be complicated by the fact that the changes in mass observed during one experiment can be attributed to conformational changes of the add-layer and/or desorption/adsorption. For this reason, QCM-D is often coupled with optical techniques, which detect only the dry mass.^204,205^ A combination of optical and piezoelectric techniques allows for water content determination and can give more detailed information on the structure of the adsorbed layer. Further development in this direction should be addressed to extensively characterize the layer structure of adsorbed enzymes. Conformational changes of adsorbed enzymes during electrocatalysis could be addressed and differentiated from film stability and protein desorption.

QCM-D constitutes a powerful tool also to perform real-time monitoring of bacterial cell deposition on a surface and biofilm growth, and it has been successfully utilized to study these processes on various inorganic materials.^206,207^ When utilizing E-QCM-D it is further possible to couple the information on biofilm formation with variations in the current–time profile. This approach has been recently utilized by Heidary et al. using *G*. *sulfurreducens* and inverse-opal ITO electrodes showing a lag phase between biofilm formation (detected by an increase in dissipation) and current generation (measured by amperometric *i*–*t* trace at +0.3 V vs SHE).^208^ The change in dissipation was utilized to monitor the variations in mass, rather than the change in frequency, due to the thick and viscoelastic nature of *G. sulfurreducens* biofilms. While the increase in dissipation occurred shortly after inoculation of the bacterial cells, the current remained almost null for more than 12 h, and significantly rose only after 4 days. Accordingly, the E-QCM-D study revealed that significant biofilm formation occurs even in the absence of EET, and that bacterial cells need to adapt to the “electrode respiring metabolism” (transfer of electrons to the electrode rather than to the natural electron acceptor).

#### 3.2.3 Atomic Force Microscopy and Scanning Electrochemical Microscopy

**Figure 7:**
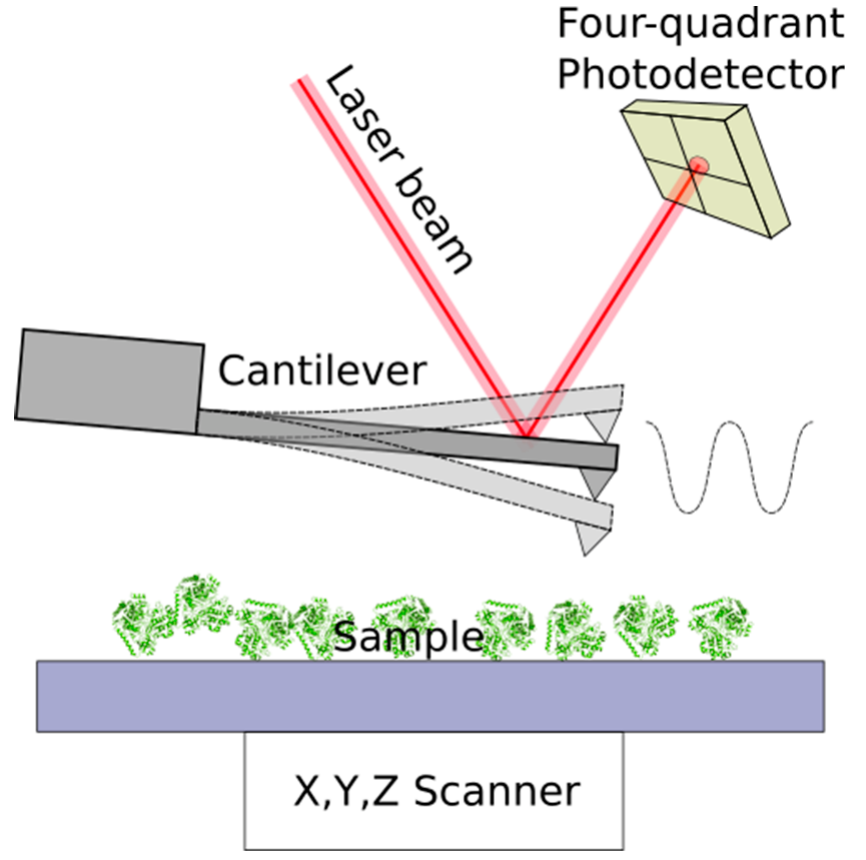
Typical setup of an AFM system. Sample is moved with a nanopositioning scanner. Tip–sample interaction deflects the cantilever, whose deformations are recorded using a laser beam reflected from the back of the cantilever onto a four-quadrant photodetector.

Atomic force microscopy (AFM) represents a well-established technique for topological investigation of surfaces and adsorbed species. Shortly, an AFM image is obtained by scanning a nanometer-sized tip across the sample. The tip is attached to an extreme of a microcantilever, which deflects as the tip interacts with the sample. Bending of the cantilever is detected by a laser beam reflected from the back of the cantilever into a position sensitive four-quadrant photodetector (Figure 7). The cantilever can be either operated in static mode (CM-AFM) or vibrated close to its resonance frequency (AM-AFM). While for CM-AFM the cantilever deflection is detected, in AM-AFM the cantilever oscillation amplitude is recorded. When the AFM tip interacts with a feature on the surface, cantilever deflection or oscillation amplitude changes. A set point value for deflection or oscillation amplitude is chosen and the tip–sample distance is reduced until the set point is reached. The topographic structure of the sample is obtained by scanning the tip over the surface and the tip–sample distance is changed to keep the set point value constant. To this end, AFM has been routinely used to address structures of proteins adsorbed on flat surfaces. High-resolution topographic images of membrane protein porin OmpF have been successfully acquired.^209^ Cysteine-modified Ompf proteins (Ompf-Cys) have been adsorbed on a flat gold surface and lateral motion of the protein has been hindered with subsequent addition of thio-lipids. High-quality images of isolated Ompf-Cys have been obtained, allowing for the trimers forming the protein to be clearly resolved.^209^

Advanced scanning techniques such as bimodal atomic force microscopy (AM-FM AFM) have enabled the accurate measurement of the elastic modulus of surfaces in liquid with subnanometer spatial resolution.^210^ AM-FM AFM has been used to simultaneously acquire the topography and the elastic modulus of adsorbed proteins.^210,211^ High-speed AFM (HS-AFM) has been used to investigate biological processes in physiological conditions with high spatial and temporal resolutions allowing for kinetic studies and real time monitoring of conformational changes. The self-assembly reaction of SAS-6 proteins to form a nine-fold radially symmetric ring structure has been successfully imaged in real-time with HS-AFM.^212^ Single-molecule kinetics of bacteriorhodopsin (BR) has been studied with HS-AFM. BR conformational changes with millisecond temporal resolution have been observed upon light irradiation activation.^213^

Extracting single enzyme activity from the macroscopic average could introduce additional understanding on how adsorbed enzymes perform their specific functionalities. For instance, the effect of enzyme conformation, orientation and surface-specific adsorption on its catalytic activity could be addressed at the single-molecule level. Scanning electrochemical microscopy (SECM) is a spatially resolved technique which uses microelectrodes to locally probe or trigger electrochemical processes on surfaces.^214,215^ Typical spatial resolution of SECM is situated in the micrometer range and, on some occasions, submicron resolution has been demonstrated,^216,217^ making this technique of particular interest for the study of microbial electrochemical systems. Bard et al. performed SECM of living bacterial cells to investigate the antibacterial effects of Ag^+^ ions on *E. coli* by monitoring the variations in oxygen concentration at the tip electrode, positioned at 25 μm from the bacterial cells, polarized at −0.8 V vs a silver paint.^218^ SECM of microbial electrodes with photosynthetic purple bacteria shed light on the capability of different diffusible redox mediators to cross one or multiple membranes of these organisms, and evidenced that the exogenous mediators react with different redox intermediates depending on the location of the bacterial cell that they can reach.^219,220^ Specifically, Cai et al. performed current–distance studies by positioning the tip electrode of the SECM on top of the bacterial cells contained in a plastic culture dish, and moving the tip closer to the bacterial cells. By fitting the obtained current–distance curves it was possible to determine the heterogeneous rate constant of electron transfer between various exogenous mediators, both hydrophobic and hydrophilic, and the redox centers of the photosynthetic bacteria. Interestingly, the study evidenced that the exogenous redox mediators react with different redox centers located in the periplasm or inside the cytoplasmic membrane depending on their hydrophilic or hydrophobic properties, respectively.^219^ Furthermore, Longobardi et al. performed SECM studies with a setup similar to that of Cai et al. but utilizing membrane fragments containing the photosynthetic apparatus of purple bacteria rather than intact bacterial cells to study the influence of the cell walls on electron transfer kinetics.^221^ The comparisons of an effective heterogeneous rate constant obtained for the same exogenous redox mediator, namely, menadione, with intact bacterial cells and isolated chromatophores were of (2.6 ± 0.2) × 10^–3^ and (4.8 ± 0.1) × 10^–3^ cm s^–1^, respectively, revealing that the diffusion of the mediator across the outer membrane strongly influences the electron transfer kinetics. SECM allowed also the real-time study of quorum sensing in aggregates of bacterial cells.^222^ Specifically, Whiteley and co-workers utilized SECM to measure the production of pyocyanin, a quorum sensing-controlled secondary metabolite produced by *Pseudomonas aeruginosa* (*P. aeruginosa*) that is important for its virulence. In their study, the tip electrode of SECM was polarized at 0 V vs Ag|AgCl to oxidize the reduced form of pyocyanin that is produced by the bacteria, determining the number of cells aggregates required for its production and the distance between different cells aggregates to stimulate the quorum sensing.

SECM studies were recently utilized also to shed light on the microbiologically influenced corrosion process of *S. oneidensis* MR-1 biofilms on stainless steel. Li et al. utilized riboflavin, an endogenous redox mediator produced by *Shewanella*, and polarized the ultramicroelectrode tip of SECM to either riboflavin reducing (−0.6 V vs Ag|AgCl) or oxidizing (−0.2 V vs Ag|AgCl) potentials, while biofilms of the bacterial cells were grown on stainless steel electrodes with or without passive layers.^223^ The study provided in situ evidence of the EET process taking place during the corrosion process, and revealed that the bacterial cells can perform bidirectional extracellular electron transfer using the diffusible mediator depending on the state of the steel surface (active or passive). As highlighted by these works, SECM studies can provide several insights on microbial electrochemical systems, conversely, when focusing on the study of single enzymes, SECM is not capable of resolving such electrocatalytic activity. To address this problem, effort has been undertaken to combine the high resolution of AFM with the electrochemical selectivity of SECM.^224,225^

**Figure 8:**
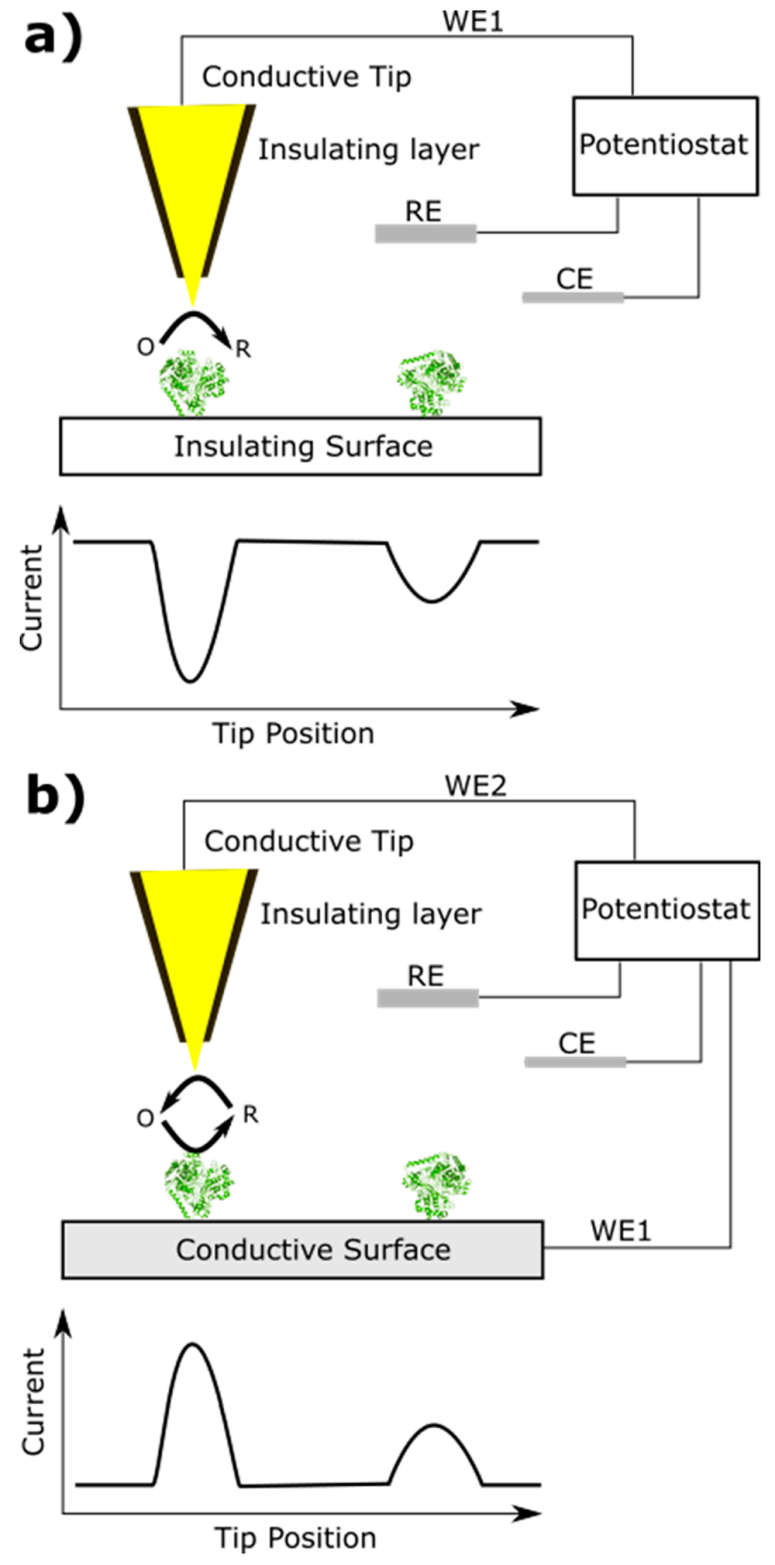
AFM-SECM working sketch. (a) Tip functions as primary working electrode (WE1). The electrochemical reaction takes place when the tip interacts with the adsorbed enzyme. (b) Tip functions as secondary working electrode (WE2). The electrochemical reaction products are detected when the tip is situated in the vicinity of the adsorbed molecule.

AFM combined with scanning electrochemical microscopy (AFM-SECM) has emerged to be a useful technique for simultaneous topographical-electrochemical measurements.^224^ In AFM-SECM, the tip can function either as primary (WE1) or secondary working electrode (WE2). As the tip is scanned over the sample, the topographic image and the electrochemical signal are recorded simultaneously. Figure 8a shows a typical setup for the probe working as primary electrode. Enzymes are adsorbed on an insulating surface. During the topographical scan, when the tip interacts with the adsorbed molecules, an electrochemical current is detected. When the tip is used as WE2, proteins are adsorbed on a conductive surface which acts as WE1. During the scan over the surface, the tip records the product of the electrochemical reaction (Figure 8b).

Local electrochemical activity sensing with a conductive probe is challenging since the cantilever is covered with a conductive layer and entirely electroactive. In such conditions, the ability to measure processes specifically at the probe-sample interface is lost due to non-faradaic currents produced at the surface of the cantilever hindering the signal originating at the apex of the probe.^226^ An ideal cantilever for AFM-SECM measurements is insulated and only the apex of the probe is conductive. Different techniques have been developed to build high-resolution AFM-SECM probes that contain well-defined electrode geometries.^226,227^ High lateral resolution of AFM-SECM has been demonstrated by imaging gold nanoparticles (20 nm) functionalized with redox-labeled PEG chains.^228^ The PEG corona and the gold core of individual nanoparticles have been simultaneously “visualized” by combining the electrochemical and topological signals. Combining scanning tunneling microscopy with SECM the catalytic turnover of hydrogenase adsorbed on gold SAM have been successfully investigated at single-molecule level.^229^ The direct observation of the single enzyme activity on the surface joined with macroscopic electrochemical measurements allowed the evaluation of a turnover frequency (TOF) for single hydrogenase molecules. Single-molecule TOF has been determined as a function of number of carbons in the SAM. When extrapolating to zero thickness, the TOF has been estimated to be ∼21,000 s^–1^ at −0.7 V vs Ag|AgCl.

Individual viral proteins marked by redox antibodies and adsorbed on virus particles have been successfully studied with AFM-SECM.^230^ Two filamentous plant viruses belonging to the Potyviridae family genus *Potyvirus* have been studied (PTV). They are rod-shaped particles (∼700–900 nm in length and ∼10–15 nm in diameter) and are made of helical winding coat proteins (CP) packing the viral genomic single-stranded RNA. An additional protein (VPg) is attached at one end of the rod-shaped particles. CP and VPg could be selectively redox immunomarked using ferrocene-functionalized antibodies. When the CP proteins were redox immunomarked, an electrochemical signal has been detected when the AFM tip was scanned over the backbone of the rod-shaped particles. Conversely, when the VPg proteins were redox immunomarked, an electrochemical current was detected only when the tip was located at one extremity of the particle.

Even though AFM-SECM has shown interesting potentials for future applications, it is still considered a highly specialized technique. The cost of production, durability, and reliability of the probes represent major challenges to improve lateral resolution necessary to achieve single enzyme activity detection. Nevertheless, the information at the single-molecule level one might obtain with AFM-SECM could lead to substantial advancements on the understanding of the electroactivity of adsorbed enzymes. For instance, correlation between electrochemical activity of adsorbed molecules and specific binding sites of the surface, such as terraces and imperfections, could drive to the development of a better electrode substrate. Comparisons of topographic heights of adsorbed protein with electrochemical currents could give insight on the preferential molecule orientation for direct electron transfer. Finally, changes in enzyme rigidity during electrochemical and electron transferring processes may provide information on the role of dynamic protein conformations.

### 3.3 Fluorescence Microscopy and Spectroscopy

**Figure 9:**
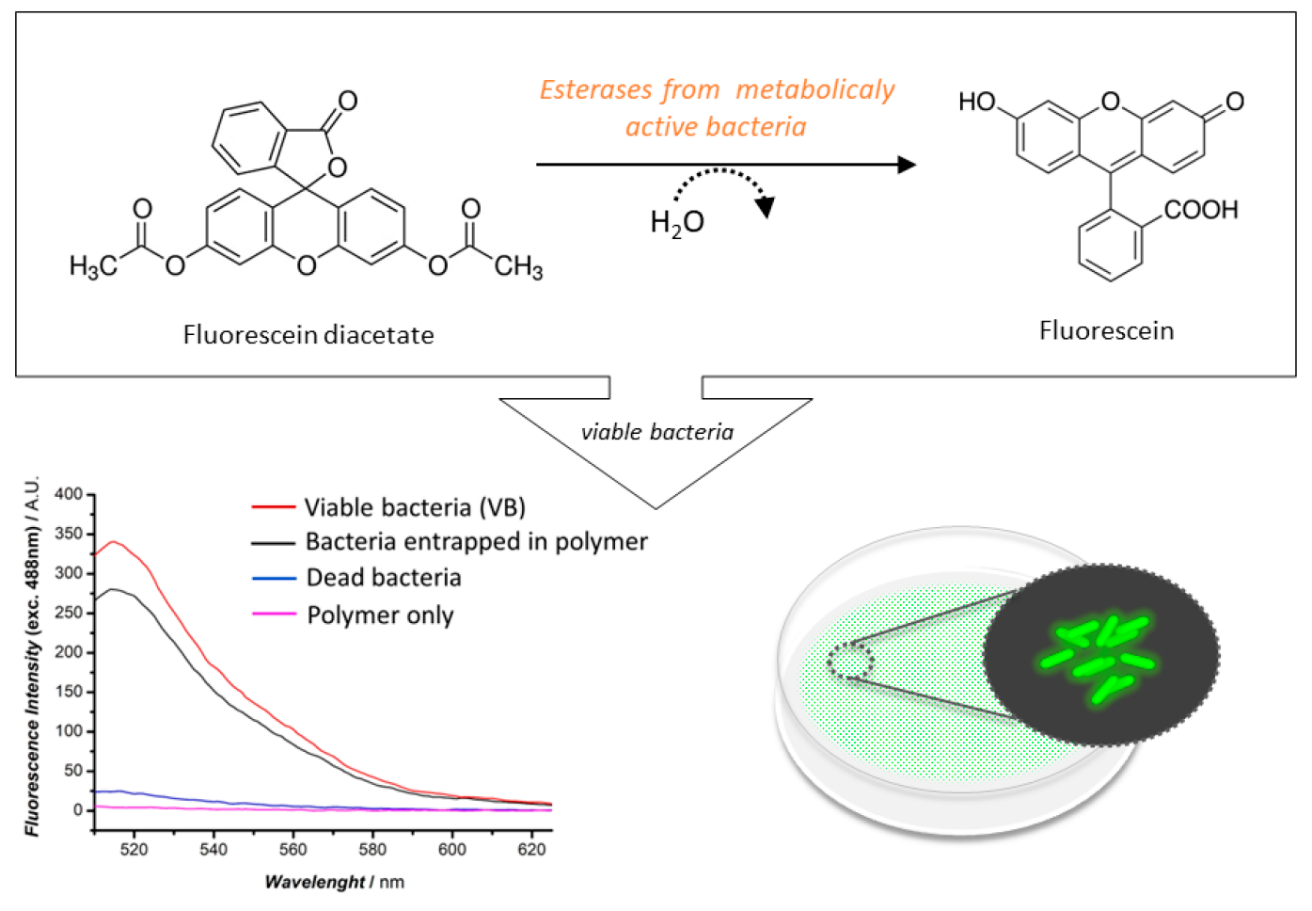
(Top) Reaction of fluorescein diacetate with esterases present intracellularly or bound to the external membrane of bacterial cells. (Bottom left) FS result obtained for free *R. capsulatus* cells (red), *R. capsulatus* cells entrapped in PDA (black), heat-treated *R. capsulatus* cells at 120 °C for 4 h (blue), and PDA only (purple), adapted with permission under a Creative Commons Attribution License from ref (81). Copyright 2022 The Authors. (Bottom right) Scheme of a microscopy slide covered in bacteria for WFM analysis.

Fluorescence microscopy (FM) and fluorescence spectroscopy (FS) are important tools for the characterization of cell physiology and morphology that can be couple to electrochemical evidence to obtain helpful information for the comprehensive study of EET phenomena. Fluorescence spectroscopy, also known as fluorimetry or spectrofluorometry, dates back to 1852 when Stokes observed the emission of light in a lower energy in relation to the wavelength at which the molecule was excited, known as the “Stokes shift”. Fluorescence rapidly turned out as a well-established method allowing the selective recognition of single cells and of specific components comprising biomolecular complex structures.^231^ FM coupled to FS plays an important role in determination of bacterial activity as it allows the deep investigation of biological processes.^232^ The development of new fluorescent probes easily adaptable to a wide array of biological applications, coupled to the technical improvements in filters, phase-contrast, and software enhanced the contrast of living microorganisms, strongly supporting the exploitation of fluorescence microscopy as a powerful research tool.^233,234^ The application of a broad range of fluorophores enables the identification of intact cells and cellular components with a high degree of specificity amidst nonfluorescing material.^235^ Wide-field fluorescence microscopy (WFM) also referred to as epifluorescence microscopy, is the most common fluorescence microscopy method used in life sciences, and it has permitted bacterial cell density and viability to be studied for the comprehension of dynamics and biogeochemical cycles in aquatic ecosystems. Specifically, in this technique the use of different fluorescent stains enables the identification and counting of cells with intact membranes (viable cells) among those with damaged membranes (nonviable) under the excitation in a specific wavelength.^236^ In microbial electrochemistry (as in enzymatic electrochemistry), the confirmation of the biotic origin of the current response plays a critical role on the study of bioelectrocatalysis. Similarly, the proper correlation of current generated and viable microbial cell loading on electrodes allows correctly comparing different electron transfer approaches by avoiding misinterpretations due to variations in cells viability. Accordingly, FM and FS provide a power tool to verify and obtain quantitative information on the viability of cells exposed to different environments or entrapped in redox polymers for facilitating EET. This approach was recently utilized by Grattieri and co-workers by combining WFM and FS to study the effects of photosynthetic purple bacteria entrapment in a redox-adhesive polydopamine matrix (PDA).^81^ Specifically, free *Rhodobacter capsulatus* (*R. capsulatus*) cells and cells entrapped in the PDA matrix were incubated with fluorescein diacetate (FDA), a colorless and nonpolar molecule. Intracellularly, or next to membrane associated esterases produced by metabolically active microorganisms, FDA is hydrolyzed to fluorescein, a green fluorescent compound (Figure 9 top), detectable spectroscopically by measuring its fluorescence. Viable and active cells are thus stained with fluorescein, while inactive cells are not stained and do not contribute to the fluorescence response. Following the incubation of *R. capsulatus* cells, their viability in the different conditions (free or entrapped) was visually confirmed by WFM and quantified by FS (Figure 9 bottom), revealing that cells retain almost 100% viability after entrapment in the PDA matrix. For comparison, cells were heat-treated to obtain dead bacteria that were also incubated with FDA, resulting in the absence of fluorescence (Figure 9, bottom, blue line).

WFM was explored also to evaluate the morphologies of anodic biofilms and their formation. It was possible to correlate the densities of mixed bacterial culture enriched at different temperatures with their power generation performance.^237^ WFM enabled continuously monitoring the different stages of grow of *P. aeruginosa* biofilms on the surface of a biosensor chip, by collecting fluorescence images at various cultivation times. Starting from the adhesion of microorganisms to the substrate surface visualized with fluorescent spots, the growth stage is determined with clusters of fluorescent areas that later reach the mature stage where homogeneous fluorescent areas are obtained, and finally the dispersal stage is revealed by a gradual decrease in fluorescence. Specifically, Liu et al. utilized carboxyfluorescein diacetate succinimidyl ester, another colorless nonpolar molecule, that, similarly to FDA, is hydrolyzed to the fluorescent carboxyfluorescein in the presence of esterases to follow biofilm formation on a microelectrode modified with Au nanoparticles, and correlated the WFM images to differential pulse voltammetry studies measuring pyocyanin production.^238^ Recently, Pirbadian et al. combined WFM with a three-electrode bioelectrochemical reactor using ITO as working electrode modified with *S. oneidensis* MR-1 cells stained with the fluorescent cationic dye thioflavin T.^239^ The charged dye worked as a membrane potential indicator, as due to its positive charge it accumulates inside negatively charged membranes. The study unveiled that when the electrode is polarized to positive potentials (+0.3 V vs Ag|AgCl) a more negative membrane potential is obtained, with an increase in thioflavin T fluorescence. Conversely, moving to a low potential polarization (−0.5 V vs Ag|AgCl) resulted in low fluorescence. In addition, the developed method allowed correlating the distance of bacterial cells from the electrode surface and EET activity, by utilizing patterned working electrodes. It was possible to rule out electron transport beyond ∼30 μm from the electrode surface, due to the matching between the cell fluorescence and the electrode pattern.

We previously presented the possibility to utilize SECM to monitor variations in pyocyanin concentration related to the number of *P. aeruginosa* cells and their distance in the work of Whiteley and co-workers, which utilized confocal scanning microscopy (CSM) to obtain a *P. aeruginosa* cell count.^222^ Compared to conventional WFM, CSM provides not only information about cell membrane integrity but also on cellular and tissues’ complex morphology and dynamics with high-quality spatial resolution and contrast. The blurred aspect of images in conventional microscopy is inherent and results from the superimposition of multiple images at the detector, which are formed at different depths of field in optical lens, depending on the focal distance adjusted. Confocal microscopes can generally be classified between noncoherent and laser microscopes. The first type uses mercury or xenon as a light source, while in the second, a single wavelength or multiwavelength laser is used to excite the sample.^240^ In confocal laser scanning microscopy (CLSM), the acquisition point by point at specific wavelengths using localized laser excitation offers advantages such as minimal background signature, versatility offered by fluorescent stains employed allowing the detection of extracellular DNA and exopolysaccharides in the samples.^241^ The ability of CLSM to collect serial optical sections from thick specimens enables 3D analysis of biofilm architecture and viability, contributing for the evaluation of efficacy and for implementation of antimicrobial drugs.^242^ CLSM was used to investigate cell attachment, biofilm formation, and spatial heterogeneity across the entire electrode surface modified with current-producing biofilms of *Marinobacter atlanticus* on a continuous flow cell. These biofilms engineered to constitutively express a chromosomal copy of the gene for the orange fluorescent protein dTomato were induced to express the yellow fluorescent protein (YFP) under continuous exposure to isopropyl β-d-1-thiogalactopyranoside (IPTG), while monitoring current generation. CLSM made it possible to observe a dynamic spatiotemporal response of YFP expression, since the fluorescence response changed according to the stage of biofilm development.^243^ A flow cell-based electrochemical reactor coupled to CLSM has been utilized also by Stöckl to simultaneously perform CLSM and electrochemical impedance spectroscopy to study the formation of electroactive biofilms of *Shewanella oneidensis* MR-1.^244^

### 3.4 Bioinformatics Analysis

Bioinformatic analysis has been gaining interest for the study of microbial electrochemical systems.^135^ This can be easily explained considering the capability of bioinformatic tools to allow studying both the genomic traits in deoxyribonucleic acid (DNA) as well as the transcription rates of ribonucleic acid (RNA). Such analysis paves the way to an unprecedented knowledge, making it possible to unveil the composition of electroactive species in mixed microbial electrodes, identifying the presence of protein-coding sequences responsible for electroactivity (i.e., presence of coding sequences for *c*-type cytochromes), or revealing how external parameters (i.e., electrode polarization, presence of toxic compounds, physicochemical conditions) influence genes expression.^245^ Specifically, whole genome sequencing allows the characterization of the entire genome of a microorganism and can be performed for multiple strains simultaneously (metagenomic). This provides quantitative information on the species present in such mixed communities, which is particularly useful to correlate electroactivity to microbial composition and study EET processes in such systems. Ishii et al. combined metagenomic analysis to transcriptomic analysis (the quantification of RNA transcripts, metatranscriptomics for mixed species communities) for electrochemically active mixed microbial communities in microbial fuel cells allowing to identify which microbial groups responded to changing electrochemical stimuli based on gene expression responses.^246^ Later, the same group utilized the combination of these two approaches to study the effect of variable electrode polarizations and substrates changes on mixed microbial consortia.^247^ The study enabled identifying the abundance of species depending on the electrochemical conditions utilized (potentials from −200 to +100 mV vs SHE), with abundance of *Geobacteraceae* microbes. Furthermore, it was shown that the different electrode polarization influenced the expression of genes related to EET, such as multiheme *c*-type cytochromes and conductive pili, revealing a remarkable capability of *Geobacteraceae* to adapt to significant changes in electrode potential and substrate availability. Recently, transcriptomic analysis was utilized to unveil the effects of salinity^248^ and changing substrates^249^ on photobioelectrocatalysis of purple bacteria. Also for these studies, the combination of bioinformatic analysis and electrochemical experimental evidence allowed to unveil the effects of external stress on the photosynthetic metabolism and resulting photocurrent production.

## 4 Conclusions

Enzymatic and microbial electrochemical systems have several exciting applications and have the potential to play a critical role in the development of sustainable bioelectrosynthetic approaches, biosensing platforms, and local micropower/decontamination facilities. However, to meet such goals, improving the fundamental understanding of the electron transfer processes at the basis of these technologies will be critical, as a detailed understanding of such processes would enable rationally designing electrodes and cell configurations for biohybrid electrochemical systems with enhanced performances. As discussed in this tutorial review paper, several approaches are now available to tune bioelectrochemical systems, from artificial electron mediation systems to synthetic biology, providing unprecedent research possibilities. At the same time, various methods to characterize in detail enzymatic and microbial electrochemical systems have been successfully applied, shedding light on the mechanisms at the basis of these technologies. It should be noted that some of the presented methods have been only recently applied to the study of bioelectrochemical systems. However, the combination of different techniques (i.e., fluorescence microscopy and SECM or CV) is providing an example of how multidisciplinary approaches are enabling a new level of understanding of these complex systems. We believe that the future research in bioelectrochemistry, with the synergistic implementation of the presented approaches and methods, will pave the way to the development of biohybrid electrochemical systems with improved performance applicable in sustainable industrial processes and everyday life.

## Notes

In this tutorial review, biological standard reduction potentials (*E*^0^′) are given at pH 7, unless stated otherwise.
